# A time series of urban extent in China using DSMP/OLS nighttime light data

**DOI:** 10.1371/journal.pone.0198189

**Published:** 2018-05-24

**Authors:** Yao Yao, Dongsheng Chen, Le Chen, Huan Wang, Qingfeng Guan

**Affiliations:** 1 School of Information Engineering, China University of Geosciences, Wuhan, Hubei province, China; 2 State Key Laboratory of Information Engineering in Surveying, Mapping and Remote Sensing, Wuhan University, Wuhan, Hubei province, China; 3 School of Geography and Planning, Sun Yat-sen University, Guangzhou, Guangdong province, China; 4 School of Remote Sensing and Information Engineering, Wuhan, Hubei province, China; West Virginia University, UNITED STATES

## Abstract

Urban extent data play an important role in urban management and urban studies, such as monitoring the process of urbanization and changes in the spatial configuration of urban areas. Traditional methods of extracting urban-extent information are primarily based on manual investigations and classifications using remote sensing images, and these methods have such problems as large costs in labor and time and low precision. This study proposes an improved, simplified and flexible method for extracting urban extents over multiple scales and the construction of spatiotemporal models using DMSP/OLS nighttime light (NTL) for practical situations. This method eliminates the regional temporal and spatial inconsistency of thresholding NTL in large-scale and multi-temporal scenes. Using this method, we have extracted the urban extents and calculated the corresponding areas on the county, municipal and provincial scales in China from 2000 to 2012. In addition, validation with the data of reference data shows that the overall accuracy (OA), Kappa and F1 Scores were 0.996, 0.793, and 0.782, respectively. We increased the spatial resolution of the urban extent to 500 m (approximately four times finer than the results of previous studies). Based on the urban extent dataset proposed above, we analyzed changes in urban extents over time and observed that urban sprawl has grown in all of the counties of China. We also identified three patterns of urban sprawl: Early Urban Growth, Constant Urban Growth and Recent Urban Growth. In addition, these trends of urban sprawl are consistent with the western, eastern and central cities of China, respectively, in terms of their spatial distribution, socioeconomic characteristics and historical background. Additionally, the urban extents display the spatial configurations of urban areas intuitively. The proposed urban extent dataset is available for download and can provide reference data and support for future studies of urbanization and urban planning.

## Introduction

Urban areas are dominated by the built environment, which includes all non-vegetation and human-constructed elements, such as roads, buildings, and runways. In this context, “dominated” implies coverage greater than 50% within a given landscape unit [1]. Urban extents draw cities’ outlines, which contain urban areas, as well as man-made vegetation and bare soil in and around urban areas. In China, with the arrival of the Lewis turning point (the structural change from an excess supply of labor to one of labor shortages) and the new normal context, the pattern of urbanization that had driven large amounts of rural labor to urban areas has disappeared [2]. Currently, the trend toward urban sprawl has been increasing. For example, hundreds of square kilometers of area have become occupied by urban development since 2000 [3, 4]. Urban extent data plays an important and basic role in the analysis of the processes and trends of urbanization.

Up to the present, a variety of attempts have been made to extract urban extents in the field of geographic information science, for example, field surveys and classifications of remote sensing images [5–9]. Field surveys involve labor-intensive methods with high costs in time and labor and cannot meet the needs of large-scale scenes of urban studies [10].

With the development of remote sensing techniques, urban studies based on satellite remote sensing images have gradually increased. Traditional methods primarily involve image classifications based on medium and high resolution data, such as TM/ETM+ images [11–13] and MODIS land cover data [1, 14, 15]. However, these data have several shortcomings. For example, it is a huge workload to process a massive amount of cloud-free data, and problems of spectral and spatial inconsistency from different images may exist. Most of the images have limited temporal resolution, which cannot meet the needs of dynamic analyses at large scales[16]. Some scholars proposed the use of DMSP/OLS nighttime light (NTL) composite products, which is stable and cloud-free with global coverage and high temporal resolution, for urban extent extraction [17–19].

The Defense Meteorological Satellite Program/Operational Linescan System (DMSP/OLS) sensor contains two spectral bands, specifically the visible/near-infrared (VNIR, 0.4–1.1 μm) and the thermal infrared (TIR, 10.5–12.6 μm) bands, which have swath widths of ~3000 km [20]. Detecting city light frequency, NTL data can indirectly show the geographical distribution of human economic activities with near global coverage. Therefore, it has become a dominant source for social and economic geography studies [18, 21–23]. For example, Li and Li (2014) used NTL data to monitor humanitarian crises [22], such as the Syrian war; Zhuo et al. (2009) used NTL data to estimate population density [24]; Falchi et al. (2016) studied light pollution and the environment [25]; and Zhao and Samson (2012) studied international economic and trade activities [26].

However, NTL data have several shortcomings. Temporally, NTL lacks continuity and comparability because of the lack of an on-board calibration system, degradation of the sensors and differences in changes among different sensors [27–29]. Spatially, NTL images exhibit blooming effects and other geometric errors because of the low spatial resolution of the OLS sensor, atmospheric effects and the accumulation of geolocation errors when mosaicking the stable NTL product (an NTL image is a mosaic of several images obtained at different times) [30]. Thus, regional differences exist in NTL data between different regions in the same year and between different years within individual regions [31, 32]. In short, NTL data cannot be used to extract dynamic information about urban sprawl directly without eliminating temporal and spatial problems [28].

Previous studies have attempted to calibrate NTL data [10, 29, 33–36]. In the temporal dimension, Elvidge et al. (2009) proposed an intercalibration method for producing a time series of NTL data based on the invariant region (IR) [36], which is currently the most widely used framework [37–39]. This method can alleviate the effects of global temporal inconsistencies using a single place and removes the global bias caused by the change in satellite sensors and sensor gain setting. However, the regional biases caused by the spatial and temporal inconsistency in atmospheric conditions and the accumulation of geolocation errors when mosaicking the NTL images cannot be removed [40, 41]. Moreover, with the continuous expansion of the urban area, the light extent can be expected to expand. And in recent years in China, the brightness value has also tended to increase. But the corrected NTL data after intercalibration may not follow this rule in practice [42–44]. Wei et al. (2014) proposed a concept of pseudo-invariant features (PIFs) as local training data and built regional linear models for temporal normalization [45]. In the spatial dimension, Sutton et al. (2001) first proposed that using NTL data for urban extraction was region-related [33]. They extracted urban extents in developing regions, developed regions and some special regions with thresholds of 40%, 80% and 90%, respectively. However, it is obvious that this method is empirical, subjective and has large uncertainties [46]. He et al. (2006) combined NTL data with urban extent statistics on the provincial scale and applied the method to a larger geographical area [35]. Many other studies have followed this approach [10, 19, 47]. Zhou et al. (2014) proposed a cluster-based model using watershed segmentation [19].

Combining the studies of Zhou et al. (2014) and Wei et al. (2014), Xie and Weng (2016) proposed a cluster-based urban thresholding method for NTL data (NTL-OUT) and got a favorable result [19], [45], [41]. However, the method has two problems: 1) The method is too complicated to be applied to a practical situation. To create a cluster mask and build a temporal normalization model for the time series of NTL, the NTL images of all years have to be summed up first. When new images are added into the time series, all clusters will change and the model has to be rebuilt, which is complex and inflexible; and 2) The method of selecting PIFs is limited. It requires the acquisition year of the ancillary reference data to be the earliest year of the study period, which increases the need for ancillary reference data.

This study proposes a pixel-based urban thresholding method for the multi-temporal and large-scale NTL data, which is more flexible and simple for practical situations, and extracts urban extents over multiple scales. We propose a universal way to segment and select the PIFs, and we constructed a temporal and spatial thresholding normalization model. A high-precision urban extent dataset for China covering the years 2000–2012 was extracted. We used ancillary test data to demonstrate the reasonableness of the results and used power function fitting for time series cluster analysis to explore the trends and pattern of the process of urbanization in China over the 12 years of the study period.

## Study area and data

**Fig 1:**
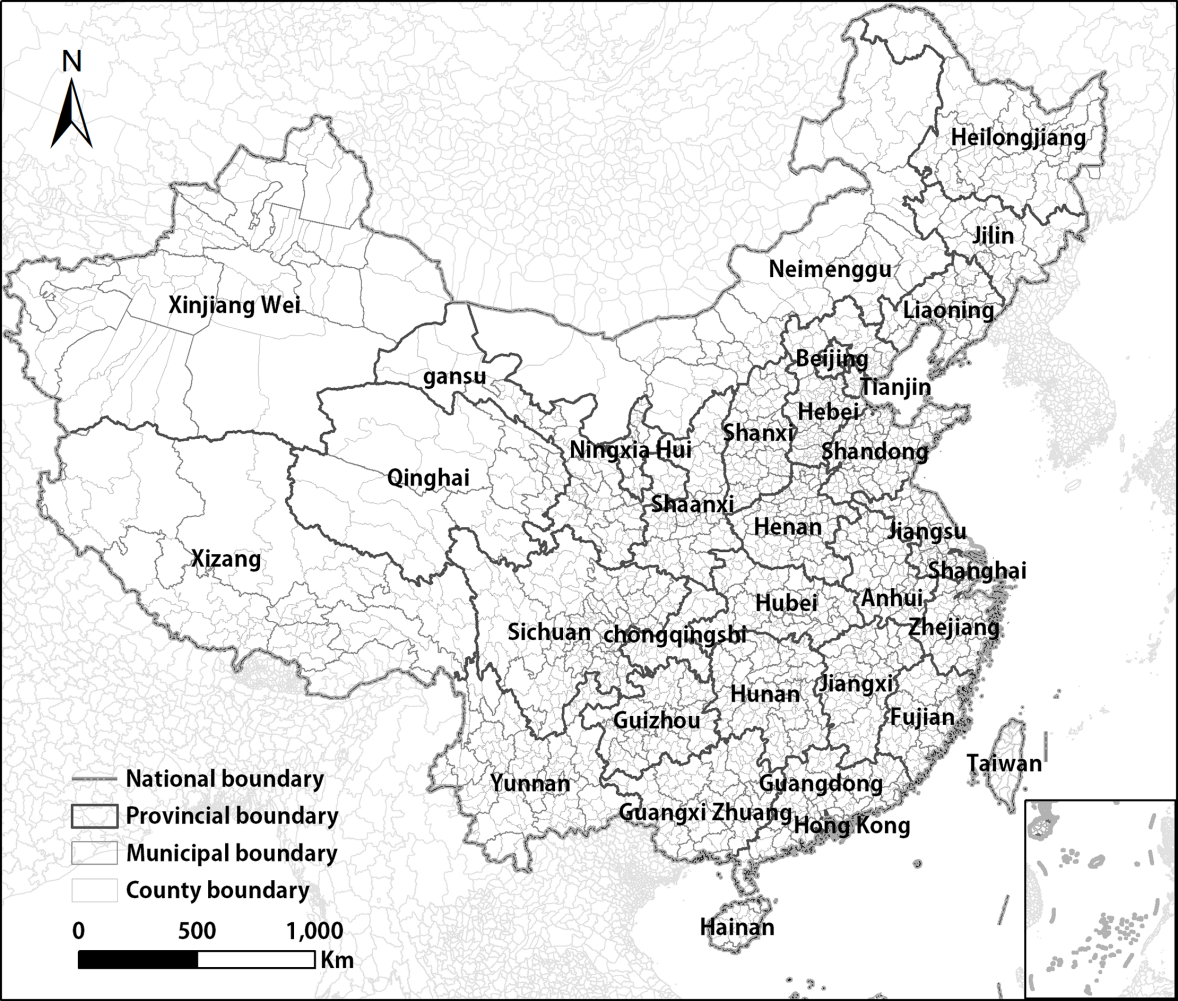
Case study area: China’s administrative boundaries at different levels.

We selected China as our study area, and the study period extends from 2000 to 2012. Since its reform and opening up, beginning in 1978, China has undergone rapid urbanization [48]. The urban area increased from 7,438 km^2^ in 1981 to 32,520.7 km^2^ in 2005 in China. The change in urban sprawl is obvious [5]. Thus, China is suitable for testing the temporal and spatial normalization model. The data on administrative boundaries in China came from the Database of Global Administrative Areas (GADM) (http://gadm.com), specifically version 2.8, which was released in 2015. The administrative boundaries stored in GADM are divided into four levels: Level 0, Level 1, Level 2, and Level 3, which address the national, provincial, municipal, and county administrative boundaries of China, respectively (Fig 1).

This study used the Version 4 stable NTL images (Version 4 DMSP-OLS Nighttime Lights Time Series; http://ngdc.noaa.gov/eog). The images between 2000 and 2012 were acquired by four DMSP satellites, F14, F15, F16 and F18. Each of these images consists of 43,201 × 16,801 floating-point values and extends from −180° to 180° in longitude and −65° to 65° in latitude. After atmospheric correction, the effects of the sun, the moon, polar light and other light were eliminated from the raw NTL data. Version 4 stable data have a spatial resolution of 30 arc-seconds and a time resolution of one year.

To extract high-precision urban extents using stable NTL images, the interference of non-urban light sources has to be eliminated first. This study uses the MOD44 W and Gas Flare masks to remove lights that appear on the water and gas flares generated by oil-collecting flare systems. The MOD44 W mask is the 250-m MODIS global surface water mask data [49], whereas the Gas Flare mask was obtained from the official NGDC website.

In this study, LULC data for China in 2010 was selected as the reference data and LULC data in 2000 was selected for data validation. LULC and their changes are important information and key parameters for studies of environmental change, geographical monitoring and sustainable development planning [50–53]. The LULC dataset for China was extracted manually from Landsat TM/ETM+ images by the Resources and Environment Data Center of the Chinese Academy of Science, and this dataset has a spatial resolution of 30 m [54, 55]. Of the 25 secondary land use types included in this dataset, urban land refers to the built-up areas in large, medium and small cities and counties, which corresponds exactly to the urban extent data that we extracted in this study [56].

In this paper, we only extracted the time series of urban extent from 2000 to 2012, because we lacked reference data and validation data prior to 2000. We will add the data from the period of 1992 to 1999 in later studies using the exact same method.

## Methodology

**Fig 2:**
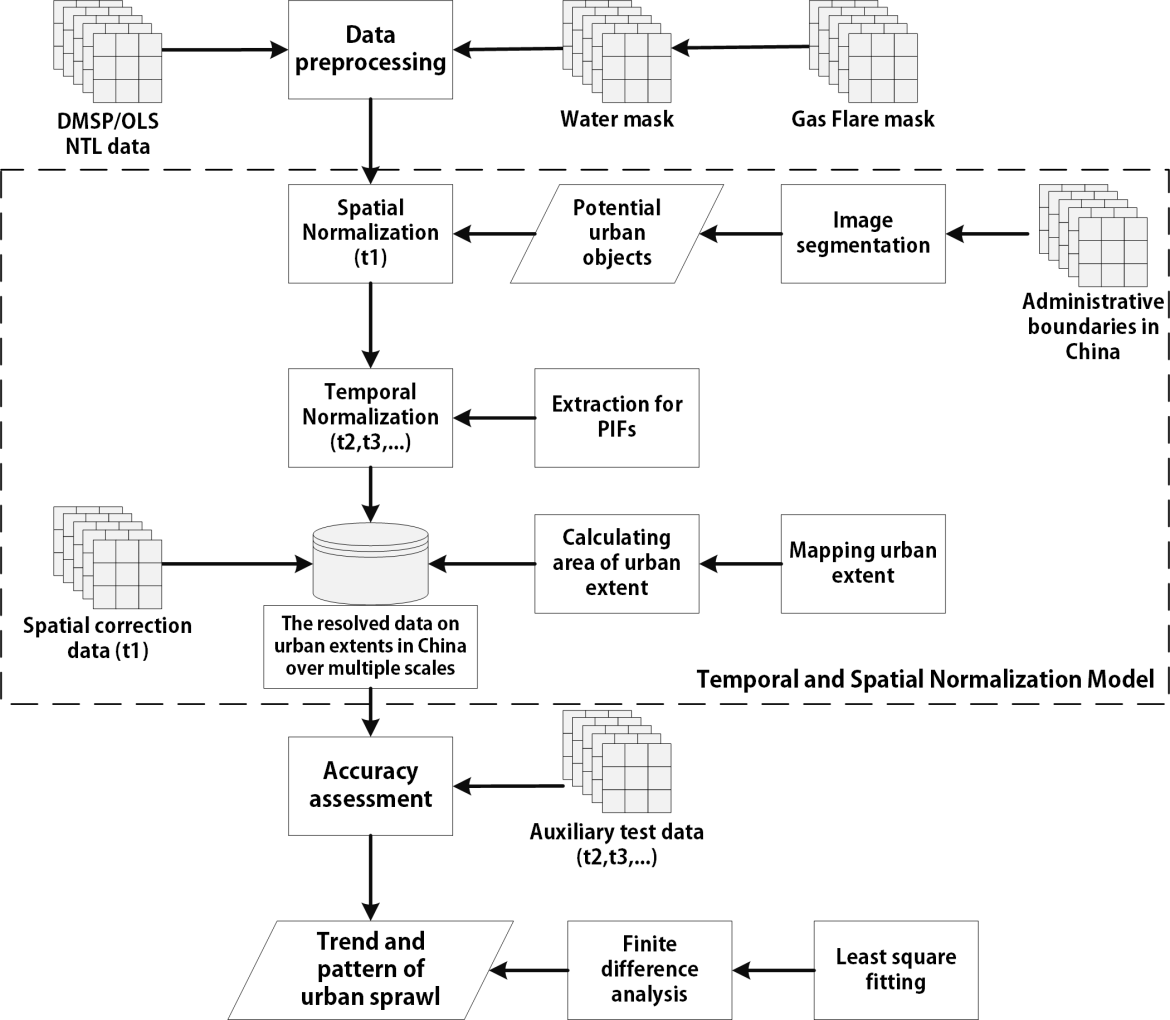
Flowchart of the proposed urban extents extraction model.

The workflow used in this study is illustrated in Fig 2. The purpose of our study is to extract an urban extent dataset covering China over multiple scales using an LULC image and time series of NTL data and to identify the different patterns of urban sprawl. In this study, we take four steps to address this problem. 1) First, we apply a spatial overlay analysis to exclude NTL data from non-city lights using the MOD44 W and Gas Flare masks. 2) Next, by comparing the NTL with the spatial correction reference data and extracting PIFs, we construct a temporal and spatial normalization model for NTL to eliminate the temporal and spatial inconsistency. In addition, we calculate the area of the urban extent on the provincial, municipal and county scales. 3) We then assess the accuracy of the results by performing validations of the urban extent dataset using the ancillary test data. 4) Finally, using the K-Means method, we cluster the extracted dataset to analyze the trends in urban sprawl in the temporal dimension.

### Data preprocessing

The NTL data are not comparable between different sensors in the same year and between different years using the same sensor, given the lack of on-board calibration on the DMSP satellites [10, 27]. The intercalibration method, a second-order regression model that is based on invariant regions across images and was proposed by Elvidge et al. (2009), is the most widely used method [36]. Using a single place as a reference, this method can alleviate the effects of global temporal inconsistency. However, the regional biases caused by spatial and temporal inconsistencies cannot be removed [30], [45]. Wei et al. (2014) proposed a concept of pseudo-invariant features (PIFs) as local training data and built regional linear models for temporal normalization [45]. In this study, intercalibration of NTL data is not needed during the preprocessing step, and we use Wei’s method to eliminate the temporal inconsistency in the next step.

As mentioned previously, further processing is required to eliminate non-urban light sources, such as the lights that appear on the water and gas flares generated by the oil-collected flare systems, from the stable NTL data. This process is carried out using the following equation [36]:

$${DN}_{Preprocessed}={DN}_{NTL}\times {DN}_{GasFlareMask}\times {DN}_{WaterMask}$$ (1)

In this study, the data preprocessing step is simple in that we only need to overlay the Gas Flare mask and the water mask on the NTL data and extract the study area from the pre-processed NTL images using the administrative boundaries. This is the basis for rapidly updating the urban extent dataset.

### Temporal and spatial normalization model

In this study, spatial inconsistency refers to inconsistencies among different regions in the same image, whereas temporal inconsistency describes a lack of comparability of measurements for the same region between NTL images obtained by different sensors in the same year or by the same sensor in different years.

To normalize the NTL data in the temporal and spatial dimensions, three steps must be followed.

Step 1 involves the segmentation of the time series of the NTL images to divide the units. We used the administrative boundaries to divide regions of NTL images, which are defined as potential urban units. It is simple and flexible for this method because when new data have to be added into the model, potential urban units do not change, and the model does not have to be reconstructed. Thus, a new result is always comparable with the original one.

Step 2 involves the spatial normalization in the reference year. Because of the regional inconsistency in the physical environment and socioeconomic conditions, the NTL DN values are not comparable across different regions [18]. The physical environment directly affects night light brightness, e.g., tall trees and atmosphere conditions, while socioeconomic conditions impact the night light fundamentally, e.g., population and economics. A single threshold cannot be used to map urban extents of different regions when the raw NTL composite products are directly used. The choices of optimal thresholds vary across regions and countries due to the regional variations in physical environments and socioeconomic development status [10, 18, 33, 35]. He et al. (2006) and Liu et al. (2012) used the region-based method using ancillary information, the administrative boundaries and the Greedy Search Method to determine the regional optimal thresholds [10, 35]. By comparing the urban extent obtained from NTL data with the urban built-up areas from the government’s statistical data, the thresholds that minimize the difference between the NTL-derived urban extent and statistical urban built-up area were selected as the optimal value through the Greedy Search Method.

In this paper, we replaced statistical data with LULC data as the spatial correction data for higher accuracy. By comparing NTL with the correction data, we searched for the optimal thresholds for all units iteratively to divide the NTL images into urban and non-urban areas. This process can be described using the following equation:

$$\sum_{n}^{i}\left|{\hat{A}}_{T,i}-A_{i}\right|=min,$$ (2)

where $\hat{A},A,n,\;and\;T$ indicate the estimated value of an urban area, the true value of the urban area, the total number of potential urban objects, and the threshold, respectively. The thresholds range from X to 63, where X indicates the minimum DN of urban lights.

In Step 3, based on the features of the NTL images in the reference and target years, robust linear regressions were identified. To train the linear regression models, this study utilized the concept of PIFs [45]. It is considered that these donut-shape polygons and non-urban areas within potential urban objects are relatively invariant regions, and the difference in DNs is caused by a temporal inconsistency. In the selection of PIFs, based on the typically irreversible nature of urbanization in recent years in China, urbanized areas without much change in nighttime light intensity during the period of the study were considered as PIFs. However, the way of selecting the PIFs based on this definition is limited, because it requires the acquisition year of the ancillary reference data to be the earliest year of the study period. Wei et al. (2014) took the earliest year among the time series as the reference year in their paper (2000). However, that method cannot work in this paper because the reference year that we define (2010) is not the earliest year (2000–2012) [45]. Therefore, we need a new method to select PIFs (Eq 3).

Our method to select PIFs is also based on the statement of the typically irreversible nature of the urbanization process. We first verified this statement in China. We counted the pixels transformed from the urban land into non-urban land using the LULC data of China in 2000 and 2010. We found that the proportion of pixels converted from the urban to non-urban areas was 0.19%. This proportion is sufficiently small that we should ignore it, and we believe that the statement can be applied to China in the recent years. Second, we built a PIF mask. We assumed that the pixels with DNs less than 63 and differences between the reference year and the target year that are less than a threshold, P, are the PIFs. The PIF mask can be described as

$$\left\{ \begin{array}{l} ({X<DN}_{2010}<63)\;\mathrm{and}\;{(X<DN}_{i}<63)\;\mathrm{and}\;(|\Delta {DN}_{i,2010}|<P),\;PIFs\\ \;Else,\;otherwise \end{array}\right.$$ (3)

where i, X, and P are the target year, the minimum DN of urban lights, and the threshold for the difference of DNs between the reference year and the target year caused by the lack of the on-board calibration, respectively. When the |ΔDN _(i, 2010)_ | is smaller than P, we consider that the actual light intensity does not change in this pixel, which is a stable lit area. Ideally, the value for P should be a value close to 0 when no temporal inconsistency exists between two NTL images.

In addition, we trained the regression models using the PIFs in the following equation:

$${PIFs}_{t2}=A*{PIFs}_{t1}+B,$$ (4)

where A and B indicate the coefficient and bias of the linear regression equation, respectively, the variables PIFs, A and B are N-dimensional vectors, and t1 and t2 indicate the reference year and target year, respectively, and N is the total number of potential urban objects.

However, the regression model cannot be trained when a potential urban object contains many saturated pixels or pixels representing non-urban lights. According to Tobler's First Law of Geography, “everything is related to everything else, but near things are more related than distant things” [57]. Therefore, we generated buffers to search for potential urban objects iteratively around the PIFs. Based on A and B, the optimal thresholds in the target year were calculated using the following equation:

$${Thresholds}_{t2}=A*{Thresholds}_{t1}+B+0.5,$$ (5)

where t1 and t2 indicate the reference year and target year, respectively, Thresholds is an N-dimensional vector.

The temporal and spatial normalization model makes the thresholds comparable with each other between the reference year and the target year. It solves the temporal and spatial inconsistency problem associated with NTL data.

Using the threshold vectors obtained from the model, the urban extents were mapped on NTL images using the equation:

$$\left\{ \begin{array}{l} {DN}_{t}\geq {Thresholds}_{t},\;urban\\ {DN}_{t}<{Thresholds}_{t},\;non‑urban \end{array}\right.$$ (6)

where t indicates the year, with a value from 2000 to 2012.

During the process of optimization and extracting the study output, we conducted a preliminary operation on the images of the urban extents and applied the sliding average algorithm to the resulting urban extent based on the statement of the typically irreversible urbanization in recent years in China, in order to remove outliers from the results. These outliers may come from such phenomena as terrain irregularities, atmospheric noise, and forest fires. These are accidental errors caused by natural and human factors. The effect was eliminated using statistical methods.

We use two primary metrics—precision and recall. Given a predicted result and a true standard, precision is the proportion of cases that the predicted result classified as positive that were positive in the true standard. It is equivalent to positive predictive value, on which Machine Learning, Data Mining and Information Retrieval all focus. Recall is the proportion of positive cases in the true standard that were classified as positive by the predicted result. It is equivalent to sensitivity [58]. Fraser Alexander & Daniel Marcu (2007) have shown that recall has a major weight in predicting the success of Word Alignment [59]. These two are often combined as their harmonic mean, known as the F-measure, which is derived from the confusion matrix and can be an equation as follows [60]:

$$F=\frac{(1+\beta^{2})\times recall\times precision}{(\beta^{2}\times recall)+precision}$$ (7)

Although between them they capture some information about the rates and kinds of errors made, these two measures and their combinations focus only on the positive examples and predictions, and ignore the information about negative cases [58]. It makes the F-measure perfect for us to use to assess the accuracy in our paper. We need to focus on the positive example (the urban extent) and reduce our attention on the negative example (the non-urban extent area), since the non-urban pixels are far more than the urban pixels. In contrast, OA and Kappa are strongly influenced by the negative example. Recall and precision are balanced when ß = 1. In most experiments, there is no particular reason to favor precision or recall, so most researchers use ß = 1 (F = F1). F1 is a constructed rate normalized to an idealized value (0–1). As the value of F1 increases, the accuracy of the result also increases.

### Analyzing urban sprawl patterns

Urban extents extracted from NTL data reflect the extent of the built-up areas. In addition, map-based representations of urban sprawl are the most intuitive reflections of the urbanization process. To compare the change in the urban extent between different objects, the ratio of urban extent change R is used, which eliminates the difference in the datum areas. This ratio can be described as

$$R_{i}=\frac{{Area}_{i}-{Area}_{2000}}{{Area}_{2000}}$$ (8)

where Area is the area contained within the urban extent and i is the year, which uses values from 2000 to 2012. Using the equation above, the curve of time series of *R* is considered to represent the pattern of urban sprawl.

Northam (1979) described the process of urbanization as an “S”-shaped curve [61]. Zhang and Seto (2011) developed five archetypes of urbanization dynamics with simulated data, including Constant Urban Activity, Early Urban Growth, De-urbanization, Constant Urban Growth, Recent Urban Growth [62]. Ma et al. (2012) applied the linear model, power-law model and exponential model with constant, convex and concave shapes to fit temporal trends of nighttime lighting areas and urbanization variables, in order to study the different patterns of urbanization processes in China [63]. According to Ma's work, we derived Eq 9 to describe the five archetypes of urban sprawl based on the curvature of curves. Areas that experienced higher levels of urbanization in earlier periods are in the Early Urban Growth class, and develop slow in late periods, whose curves exhibit a convex temporal shape. In contrast, the Recent Urban Growth class has a concave shape. This process can be described as

$$y=a+b\times x^{c}\left\{ \begin{array}{l} b>0,\;0<c<1,\;Early\;Urban\;Growth\\ b>0,\;c=1,\;Constant\;Urban\;Growth\\ b>0,\;c>1,\;Recent\;Urban\;Growth\\ b=0,\;Constant\;Urban\;activity\\ b<0,\;De-urbanization \end{array}\right.$$ (9)

where x, y indicates time, and the area within the urban extent *R*, respectively.

To fitting the time curve of *R* mentioned above with the power function, least squares fitting was used. RMSE was used to assess the accuracy of the fitting results.

To further analyze the clustering result, the ratio of urban extent change, R, is derived at time t. Since the clustering results are discrete, we used the finite difference method. The finite difference method (FDM) divides the continuous time into several meshes by time step using the Taylor series expansion, and uses a function to approximately replace the derivatives of the differential equation with the difference between the mesh junctions (nodes). The first derivative can be described as:

$$\frac{\partial R}{\partial t}=\frac{R_{t+1}-R_{t-1}}{2}$$ (10)

The equation’s physical meaning is the rate of increase of urban sprawl. Values greater than 0 indicate that the urban area is growing, and the greater the value is, the greater is the rate. It remains steady at 0 when the area of urbanized land is stable. The second derivative equation is

$$\frac{\partial^{2}R}{\partial t^{2}}=\frac{R_{t+1}+R_{t-1}-2\times R_{t}}{4}$$ (11)

Its physical meaning is the acceleration of the urban sprawl. Positive values indicate that the urban sprawl is increasing in size at an increasing rate, whereas 0 indicates uniform growth. Therefore, the growth pattern is determined according to the sign of the rate and acceleration.

## Results

### Extracted urban extents in China

**Fig 3:**
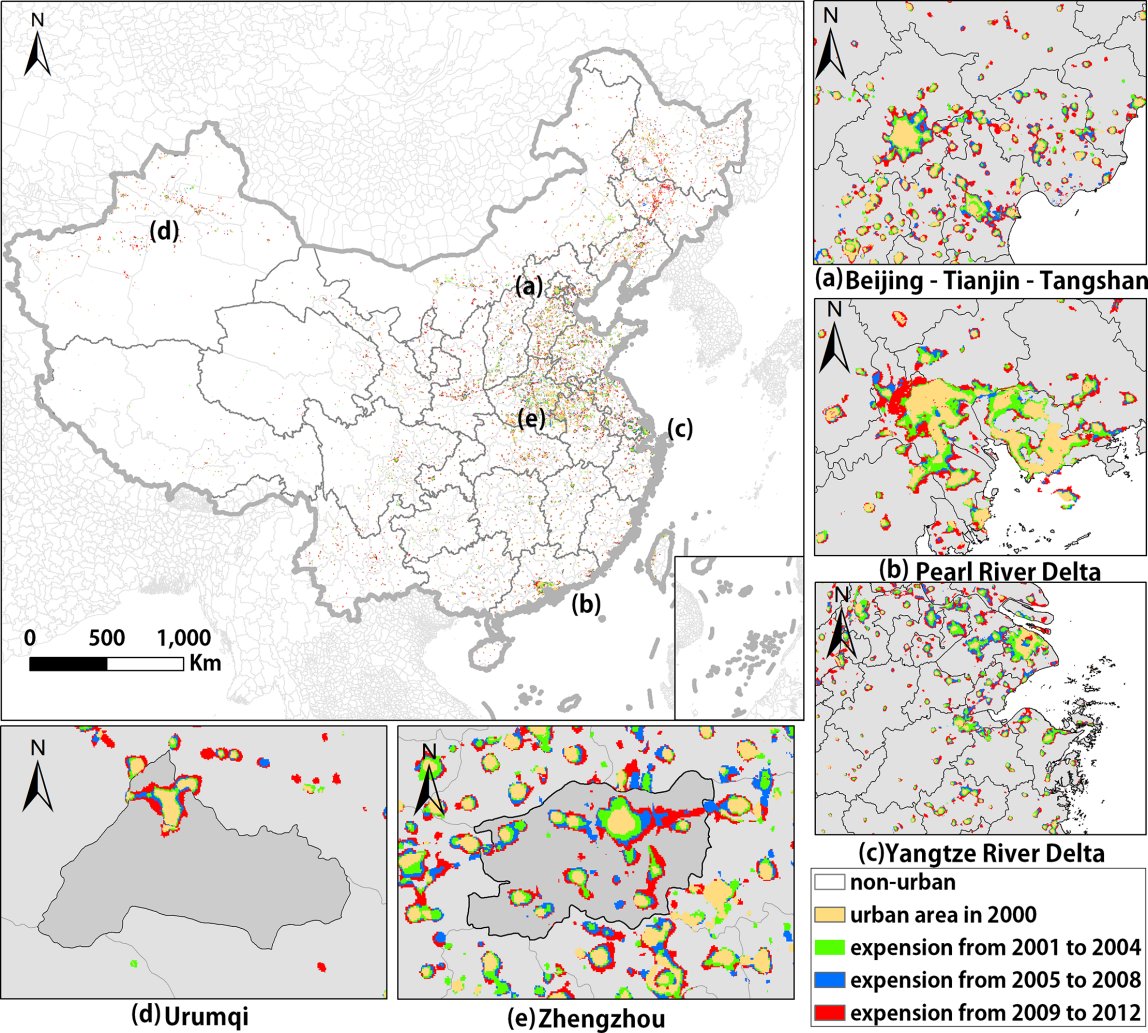
Urban extents of cities in China extracted in this study. (a) the Beijing-Tianjin-Tangshan district, (b) the Pearl River Delta, (c) the Yangtze River Delta, (d) Urumqi in the Xinjiang Uygur Autonomous Region, (e) Zhengzhou in Henan Province.

Our study used 2010 as the reference year and the other years as target years, and extracted a dataset of urban extents in China from 2000 to 2012 (Fig 3).

Using the method this paper proposed, we extracted the time series of urban extents (Fig 3), and an accuracy assessment was conducted to compare the result with the urban map of LULC. The accuracy of some major cities across this country is shown on Table 1. The OA, Kappa and F1 Score were 0.981, 0.774, and 0.782 respectively. The F1 Score excludes non-urban pixels out of the equation, focusing on urban pixels.

**Table 1:** Accuracy assessment of urban extents in 2000 extracted at county scale.

	OA	Kappa	F1
**China**	0.981	0.774	0.782
**Beijing**	0.961	0.625	0.663
**Shenzhen**	0.918	0.502	0.616
**Guangzhou**	0.904	0.539	0.592
**Lhasa**	0.999	0.608	0.642
**Urumqi**	0.985	0.632	0.601
**Jiayuguan**	0.988	0.837	0.843
**Haikou**	0.967	0.577	0.584
**Nanyang**	0.988	0.889	0.899
**Nanning**	0.990	0.519	0.507
**Wuhan**	0.951	0.534	0.562
**Changchun**	0.960	0.506	0.539

In addition, we used the administrative boundaries of different levels to divide the NTL units, conducted this method at different scales to extract urban extents, and assessed their accuracy (Table 2). From the provincial scale to the county scale, the OAs were 0.931, 0.981, and 0.996 respectively, whereas the Kappas were 0. 602, 0.774 and 0. 793. The results showed that as the scale decreases, the OA and Kappa all increase. Theoretically, the smaller the scale the images are segmented on, the more temporal and spatial normalization models are built. Thus, each potential urban object will contain a greater number of pixels with similar NTL intensities, and the estimated values will be closer to the truth. In short, when the model is built on the county scale, the smallest scale, the highest-precision results are obtained.

**Table 2:** Accuracy assessment of the extracted urban extents at the county, municipal, and provincial scales in 2000.

	County scale	Municipal scale	Provincial scale
**OA**	0.996	0.981	0.931
**Kappa**	0.793	0.774	0.602

**Fig 4:**
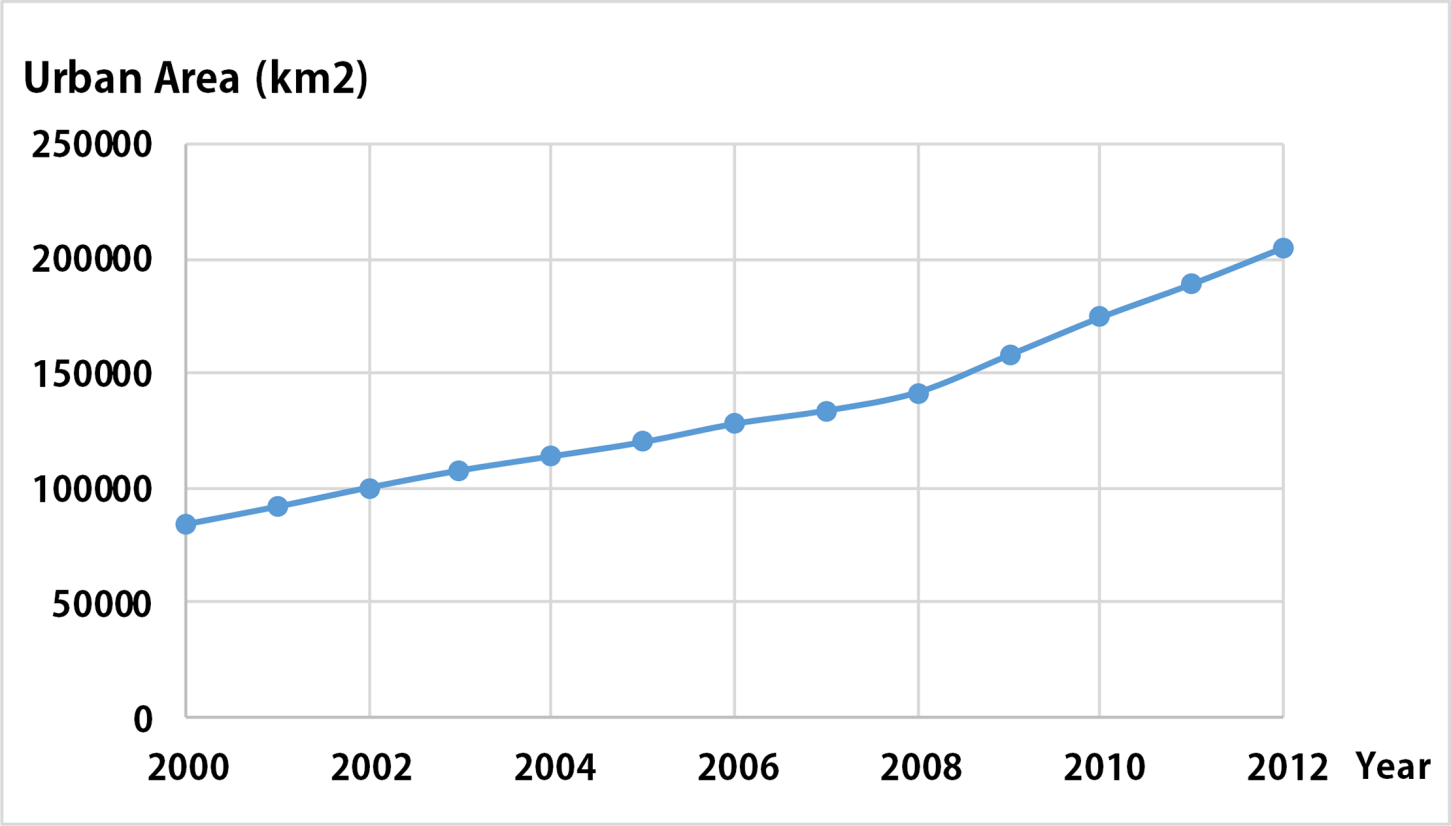
Trend of the urban area series in China.

We counted the urban area of China in 2000–2012 (Fig 4). The trend of the urban area series in China increases year by year, similar to the trend of average DN values from corrected NTL data on China by Liu et al. (2012)[10]. Our method can eliminate the regional spatial and temporal inconsistencies in atmospheric conditions and the accumulation of geolocation errors when mosaicking the NTL images. On one hand, the effects of spatial inconsistency between different regions in the same year were largely eliminated when the result in the reference year was directly compared with the LULC data. On the other hand, our method has eliminated the temporal inconsistency for the results in the target years after PIFs were used to train the models.

### Uncertainty analysis of the extracted urban extents

Since the NTL data contain non-urban lights from agricultural regions or regions with low population densities, Zhao et al. (2015) and Xie and Weng (2016) used a threshold of 5 to filter out non-urban lights during the process of segmentation, because these pixels had a lower possibility to belong to urban areas in China [41, 44]. Therefore, in this study, we chose 5 as the minimum DN value for the urban areas. During the process of iterative calculation in the spatial normalization, non-urban pixels were directly ignored.

**Fig 5:**
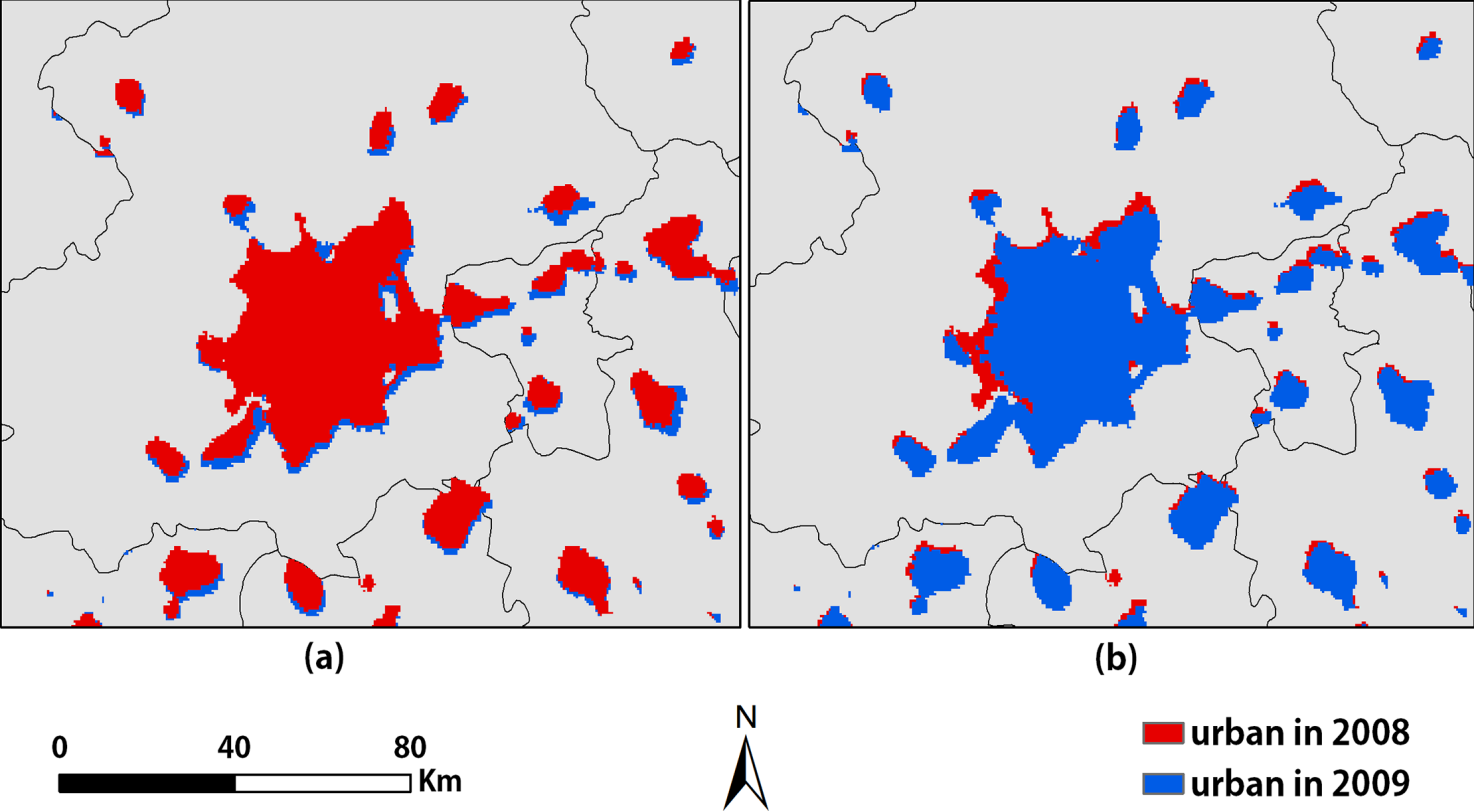
Offset in the extracted urban extents of Beijing in 2008 and 2009. (a) The result for 2008 is superimposed on the result for 2009; (b) The result for 2009 is superimposed on the result for 2008.

A slight spatial offset exists between different years in the same region. The urban extent in 2008 is slightly offset to the northwest relative to the result in 2009 (Fig 5(A) and 5(B)). Although this offset is not obvious, it affected the selection of PIFs and the accuracy of the process of the temporal normalization to some extent. This is due to the influence of many factors, such as the low spatial resolution of the OLS sensor, atmospheric effects and the accumulation of geolocation errors when mosaicking the NTL images [30]. The urban lights do not exactly coincide geographically between the NTL data from different years, and the urban extents obtained using NTL data are offset from the built-up areas generated using Landsat TM images [64]. Therefore, we calculated the average images using the NTL images obtained by different sensors in the same year, which can reduce the impact of the offset to a certain extent. For example, data from 2000 to 2003 were the average of NTL images obtained by F14 and F15.

Xie and Weng (2016) found that the results of linear regression were insensitive to the selection of PIFs when the NTL intensity uniformity within the same object is sufficiently high [41]. When the scale of image segmentation is small enough, it is concluded that the pixels within the object are similar.

**Fig 6:**
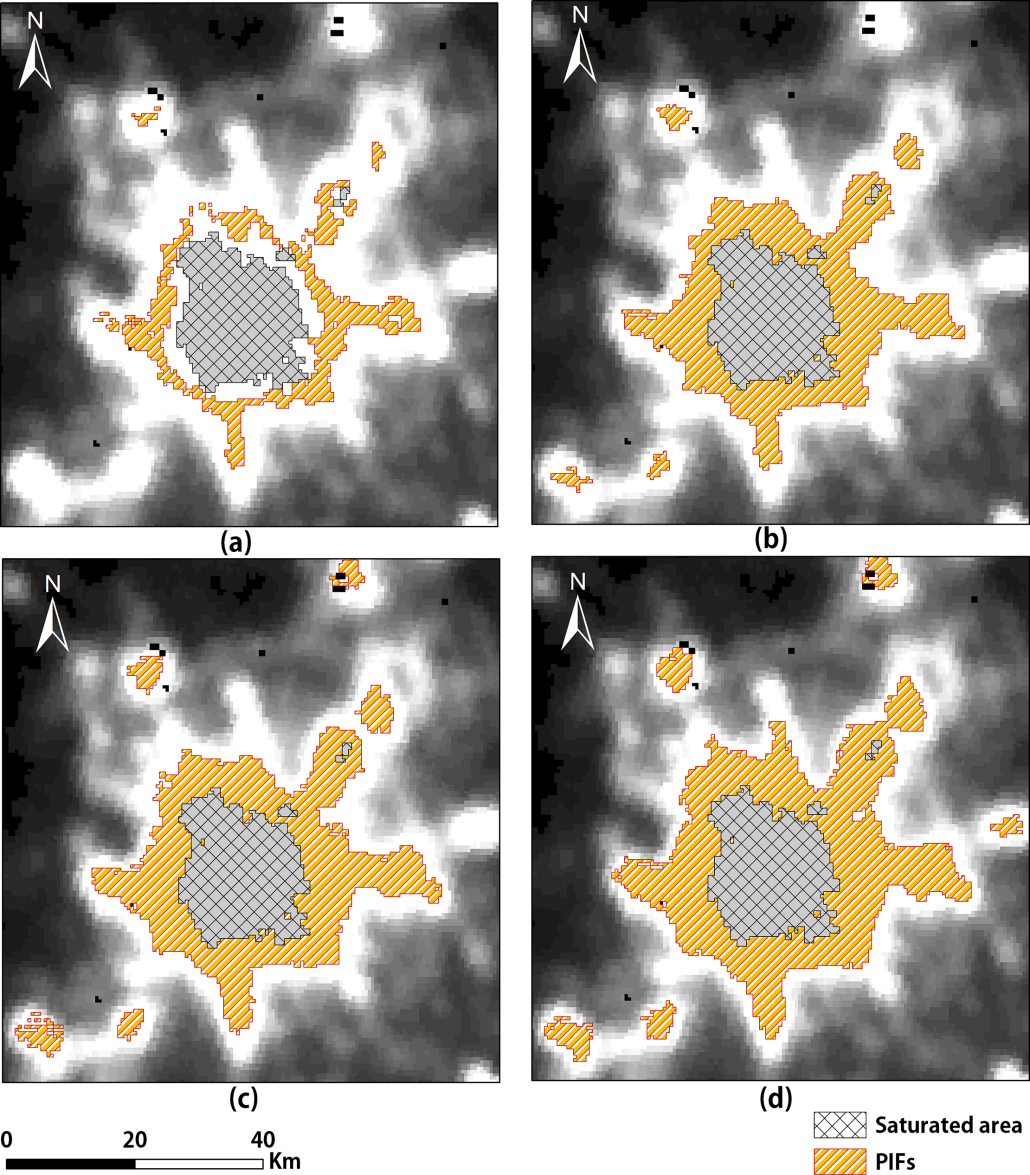
Selection of PIFs. (a) The difference is not more than 1; (b) The difference is not more than 3; (c) The difference is not more than 5; (d) The difference is not more than 7.

As the value of P increased, we found that the area of urban sprawl (the orange area) and the non-urban area became larger (Fig 6). When *P* is too large, the expansion of urban sprawl is more likely to be considered as representing PIFs (Table 3). When *P* = 5, OA reaches 0.996, and the Kappa achieves the largest value. In summary, this study selected pixels with DN changes less than 5 as the PIFs to train regression models on a small scale. In addition, the regression models cannot be constructed for potential urban objects that contain no PIFs. To make sure that all units get PIFs for training models, according to Tobler's First Law of Geography [57], this study instead generated buffers of 15 pixels to search the surrounding PIFs iteratively. There were 15 pixels derived from experience to guarantee that all units could get PIFs and that the model could run effectively at the same time.

**Table 3:** Accuracy assessment of the extracted urban extents using different P values in 2000.

	P = 1	P = 2	P = 3	P = 4	P = 5	P = 6	P = 7
**OA**	0.993	0.991	0.992	0.993	0.996	0.992	0.992
**Kappa**	0.522	0.345	0.419	0.601	0.789	0.731	0.721

**Fig 7:**
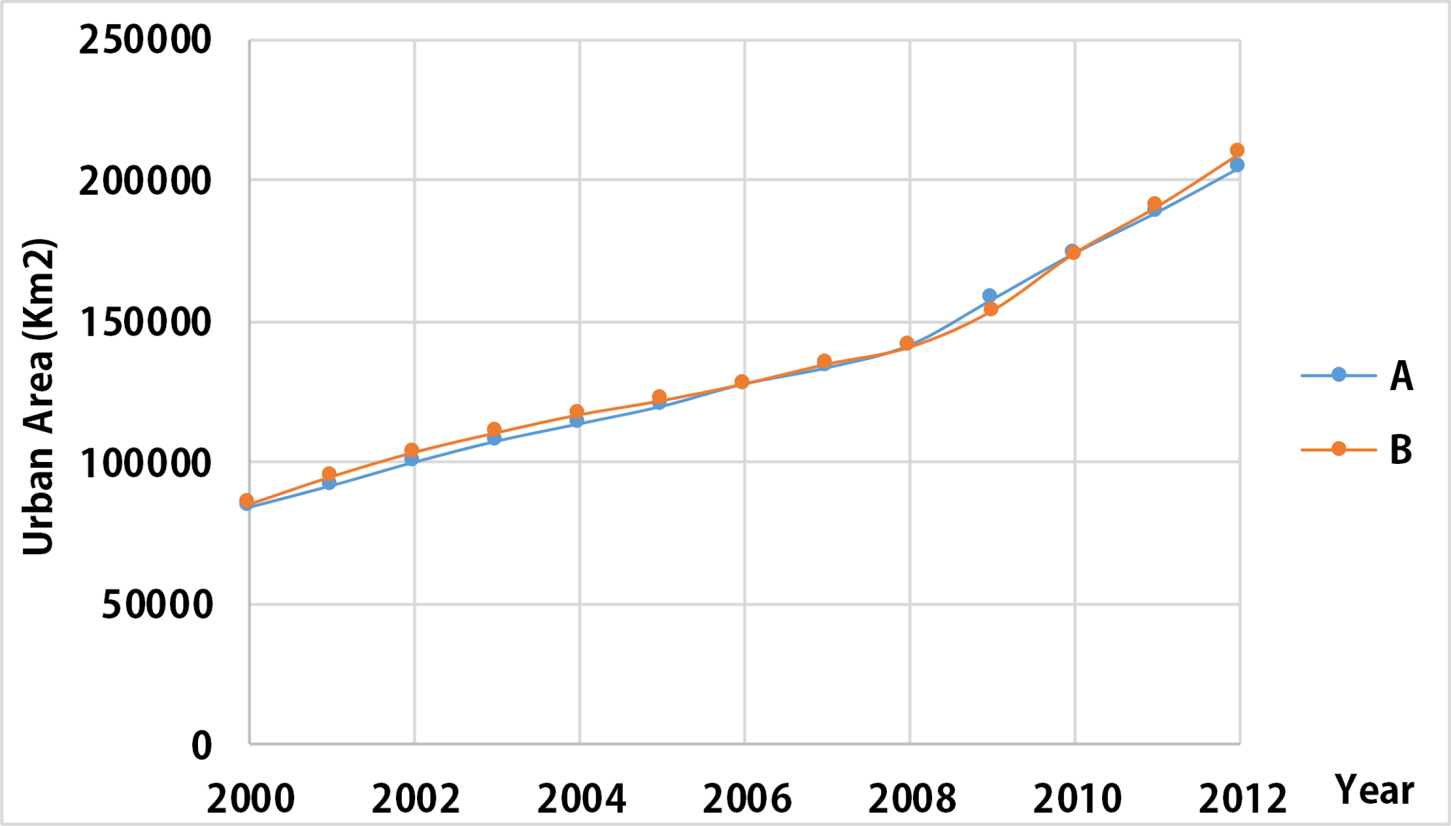
Trend of the urban area series in China. **(**A) The urban area using the raw NTL dataset and the proposed temporal and spatial normalization; (B) The urban area using the intercalibrated NTL dataset and the proposed temporal and spatial normalization.

In order to prove that the proposed method is able to alleviate the temporal inconsistency of DMSP/OLS NTL data, we conducted an experiment. First, we followed the works of Elvidge et al. (2009) and Liu et al. (2012) and used the intercalibration method and intra-annual composition method on DMSP/OLS NTL data, taking City of Jixi as the calibration area and 2007 nighttime light data from satellite F16 as the reference dataset [10, 36]. And then we applied the intercalibrated NTL dataset to the proposed temporal and spatial normalization model and obtained the new time series of urban extent. At last, we compared the new dataset of urban extent with the original time series one in the trend toward the urban area and accuracy assessment (Fig 7, Table 4).

**Table 4:** Accuracy assessment of the extracted urban extent at the county in 2000 using different datasets.

	The raw DMSP/OLS dataset	The intercalibrated NTL dataset
**OA**	0.996	0.996
**Kappa**	0.793	0.790
**F1-Score**	0.782	0.783

Table 4 shows that slight differences exist when we use different NTL datasets (the raw NTL dataset and the intercalibrated NTL dataset). The F1-Score of A is higher than B, while the Kappa of A is lower than B (A, the urban area using the raw NTL dataset and the proposed temporal and spatial normalization; B, the urban area using the intercalibrated NTL dataset and the proposed temporal and spatial normalization.). However, the differences between A and B are sufficiently small that we should ignore it (0.001~0.003). Therefore, we consider that there is no difference of using the intercalibration method or not. We believe that it is the cause of the PIFs-based linear regression that Wei et al. (2014) proposed, which is applied in the proposed temporal and spatial normalization [45]. Therefore, the temporal and spatial normalization is able to address the problem of the comparable DN values of DMSP/OLS among years.

### Analyzing urban sprawl trends in China

In this section, we used power function fitting of the temporal curves of urban sprawl at the county scale, and classified the archetype of the urban sprawl pattern, depending on the parameters of the function. In addition, the finite difference method was used to calculate the rate and acceleration of the urban sprawl for fine analysis on some regions.

**Fig 8:**
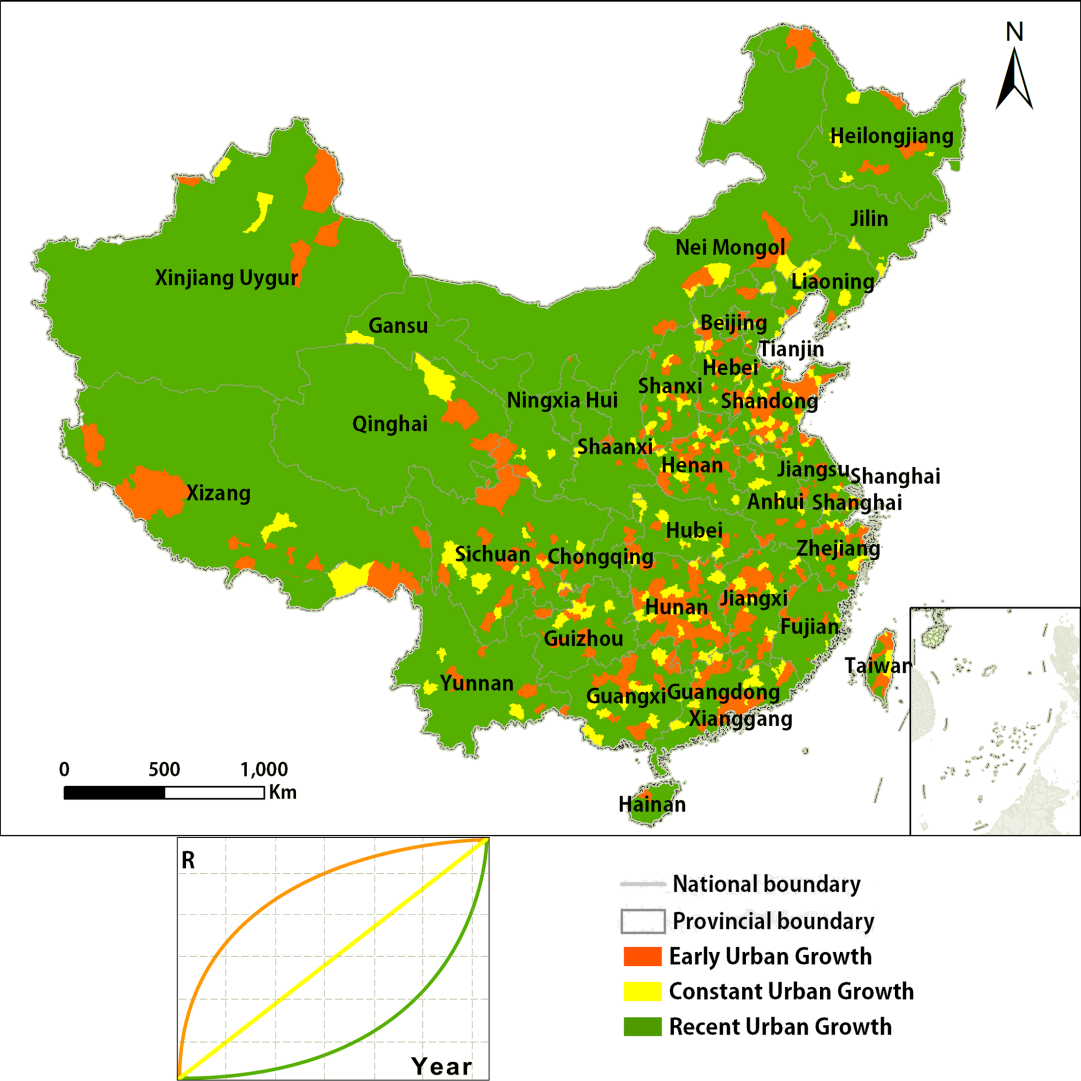
Spatial distribution of analysis on the urban sprawl pattern in China.

The spatial distribution of the urban sprawl pattern displays some aggregation, which is approximately distributed in the eastern, central and western areas (Fig 8). De-urbanization and Constant Urban Activity do not exit or have not appeared in China. Only three patterns of urban growth are shown on the map. Early Urban Growth is mainly observed on the eastern coast in places such as Guangdong, the Yangtze River Delta, and Beijing. These areas developed early and most of them are economically developed areas at present. We can describe these areas at the end of the “S”-curve of urbanization, which develop fast early, and slow down later [61].

Most of the counties in China belong to the type of Recent Urban Growth. Their urban area does not change much at first, but grows fast in recent years, with the largest growth rate and acceleration. Therefore, the pattern of Recent Urban Growth is in the early stages of the “S”-curve of urbanization. Meanwhile, some counties belong to Constant Urban Growth, which means they are in the middle stage of the “S”-curve. Considering the above, the urban sprawl can reflect the process of land urbanization.

The result of the classification is consistent with the socioeconomic growth of the western, eastern, and central regions. The reason may be related to the implementation of the western development strategy and the effect of the global financial crisis in 2008. Further study is required to determine the exact reason.

**Fig 9:**
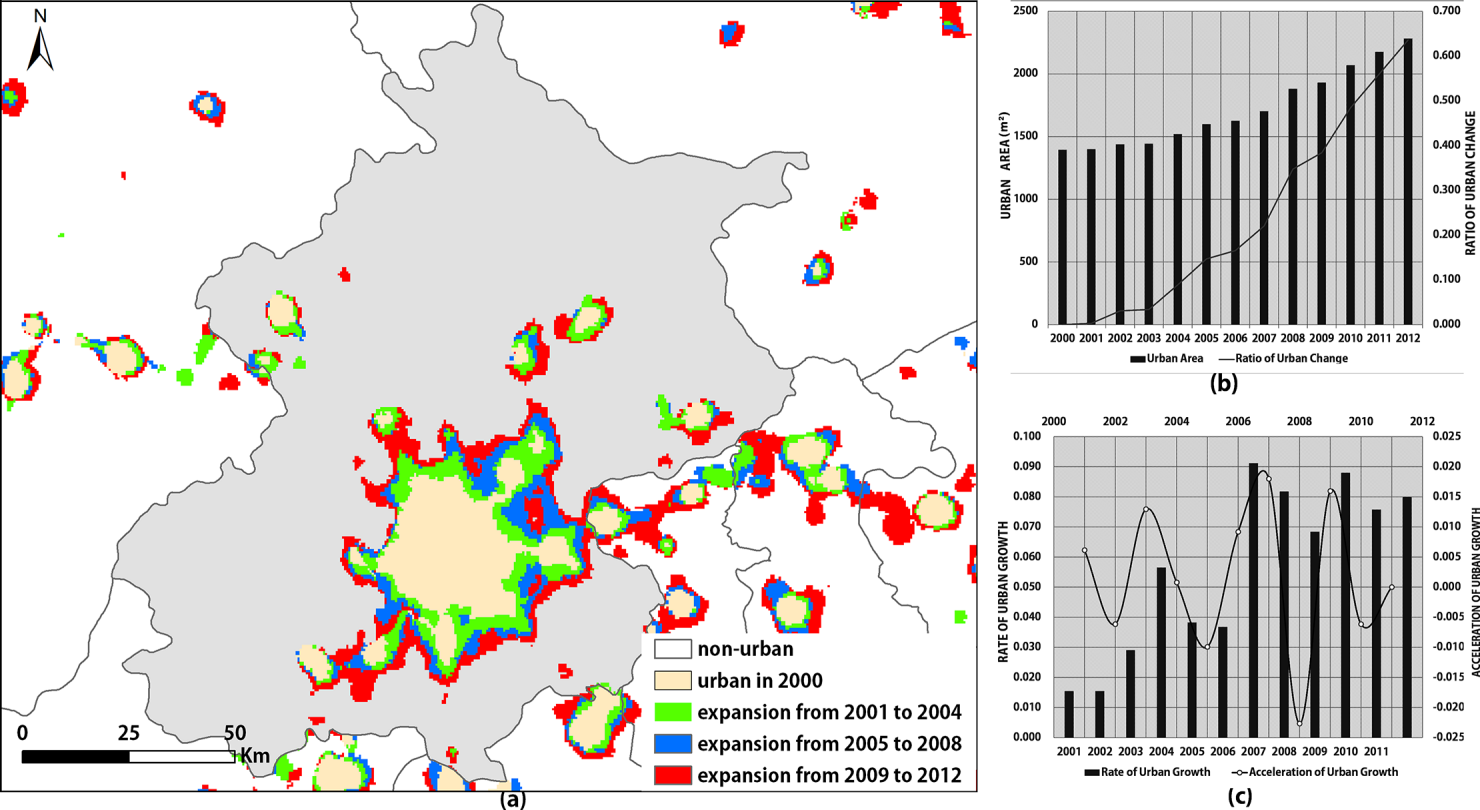
Analysis of urban sprawl in Beijing. (a) The urban extents in Beijing; (b) Curves displaying the area and ratio of urban sprawl in Beijing; (c) Curves displaying the rate and acceleration of urban sprawl in Beijing.

We identified two noteworthy points in Fig 9: 1) The spatial configuration of the urban areas in Beijing is representative of the typical satellite-town model, which is one of the host-satellite clustered city models. Since 1990, Beijing has attached great importance to the construction of satellite towns to promote the socioeconomic development of the surrounding regions; 2) The spatial configuration of the central regions reveals the concentric zone model, in which the center sprawls outwards following a concentric circular pattern and ring roads follow the shape of concentric circles [40]. In addition, as urban sprawl progresses, the urban area in the center gradually merges with those of the surrounding satellite towns, thus realizing the integration of the urban infrastructure. This is a typical pattern of urbanization in China [65].

**Fig 10:**
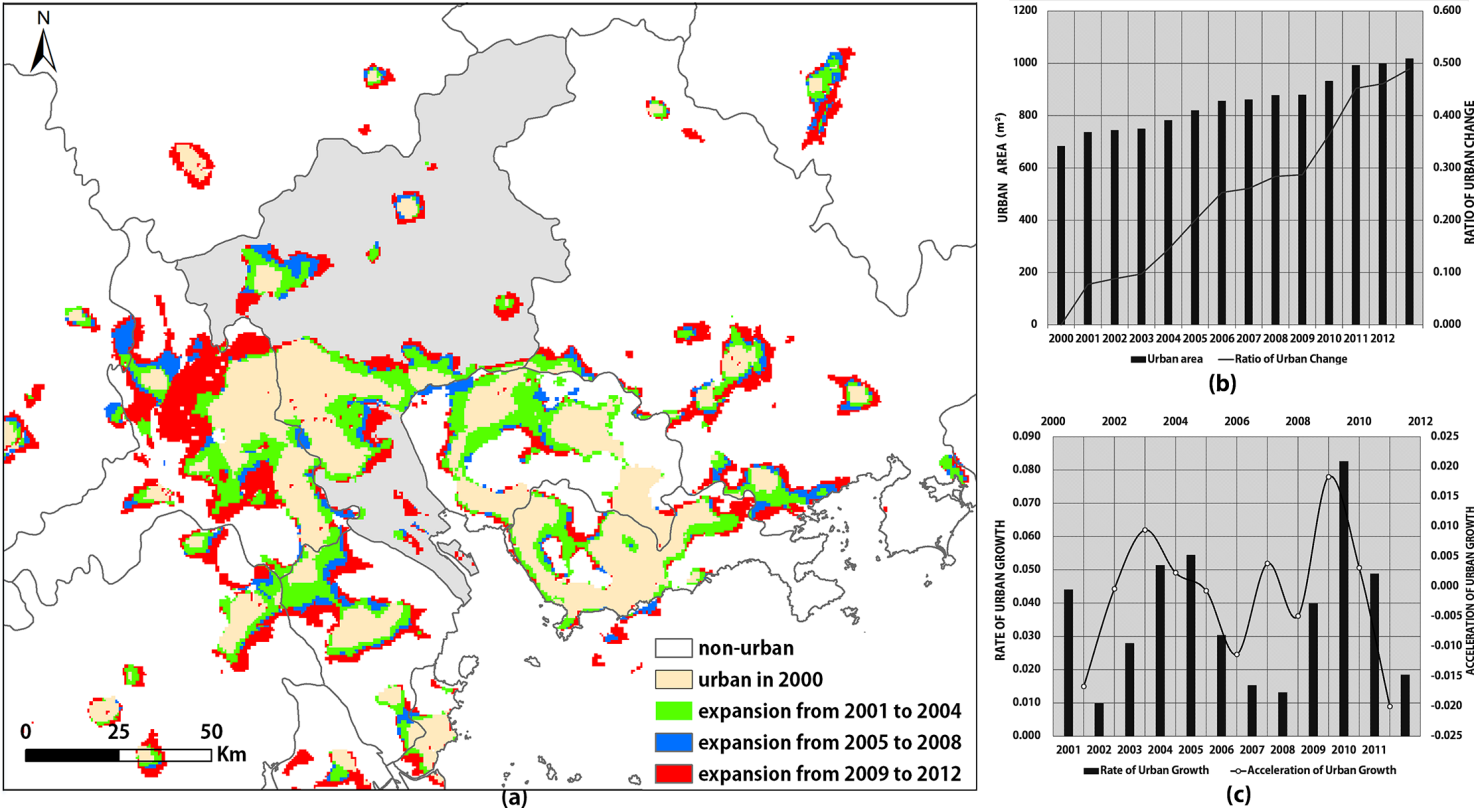
Analysis of urban sprawl in Guangzhou. (a) The urban extents in Guangzhou; (b) Curves displaying the area and ratio of urban sprawl in Guangzhou, (c) Curves displaying the rate and acceleration of urban sprawl in Guangzhou.

The spatial configuration of the city of Guangzhou and its surrounding towns represents a megalopolis; it has a multi-core structure and each of these cores has complete functions, and the region serves as an economic hub. [66]. The expansion and connection of the urban extents are steadily progressing (Fig 10(B) and 10(C)). Since 2000, the urban extents in Guangzhou have been bordered by the surrounding cities, especially in the eastern part of Foshan Shunde and northern Zhongshan. In addition, Le et al. (2016) believed that the construction of the traffic infrastructure between the two cities can improve the convenience of investment and production of enterprises in both places and can also reduce the commuting cost to consumers and promote consumption in both places [67]. Therefore, the construction of the second phase of the Guangzhou-Zhuhai West Line in 2005 and the opening of the Zhongshan Northern Urban Railroad Station in 2011 have, in fact, promoted industrial linkages between Guangzhou, Foshan, Shunde and northern Zhongshan, and has accelerated the integration of their urban areas.

## Discussion and conclusions

To extract a time series of urban extents from DMSP/OLS NTL data using the thresholding method, two problems need to be solved: First, because of noise from non-urban light sources, the low spatial resolution of the OLS sensor, atmospheric effects and the accumulation of geolocation errors when mosaicking the NTL images, NTL data cannot be used directly to generate large-scale scenes of urban areas. Second, the lack of an on-board calibration system causes NTL images to not be comparable with each other. Therefore, given the temporal and spatial inconsistency of NTL data, they cannot be directly used to extract urban extents.

This study constructed an improved and simplified spatiotemporal thresholding normalization model for a practical situation at the pixel level. We used image segmentation, linear regression and PIFs to process the NTL data. This model effectively and quickly extracted the urban extents on county, municipal and provincial scales. The construction of the space-time normalization model requires three steps. 1) First, potential urban objects are extracted using the pre-processed NTL dataset and the administrative boundary data using image segmentation. 2) Next, in the reference year, the iterative searching method was used to compare the NTL data with the LULC data to eliminate the spatial inconsistency. 3) Finally, based on the PIF and linear regression methods, the temporal normalization model between the reference year and the target year was constructed to eliminate the temporal inconsistency. In addition, an accuracy assessment leads to the following conclusions: 1) The smaller the units on which the model is constructed, the higher is the accuracy. 2) In the target years, the values of OA, Kappa and F1 Score demonstrate the high accuracy of the dataset.

The method proposed in this study has several advantages. 1) Since this method does not involve intercalibration of the NTL data during the pre-processing step, the method can greatly reduce the amount of cumbersome pre-processing required and improve the degree of automation of the program; 2) The selection of the reference year is limitless because we proposed a flexible way to select PIFs. This feature greatly reduces the requirements for ancillary reference data and effectively shortens the calculation time; 3) When new images are added into the time series, the units will not change because we used the administrative boundaries to divide NTL units and built the model at the regional level, which makes the different result using the different dataset comparable; 4) With the limitation of DMSP/OLS’s spatial resolution (1 km), previous studies exported the urban extent with 1-km resolution [10, 19, 35]. We increased the spatial resolution of the urban extent to 500 m (about four times finer than before). We resampled the LULC to 500 m. With the limitation of LULC, the lights in the water and vegetation inside the city were removed. In addition, the temporal resolution of the result is appropriate using the annual NTL composites product. In conclusion, this improved and flexible method is much easier to be applied to the practical situation than the NTL-OUT method, and we can also get datasets of urban extents with high precision.

In addition, the extracted urban extent dataset produced by this study was used for pattern analysis. Moreover, we identified the urban sprawl trend of China's counties from 2000 to 2012. 1) All counties of China display a growth in urban sprawl from 2000 to 2012. It means that De-urbanization and Constant Urban Activity did not exist in China during 2000 to 2012. 2) According to the convexity of the temporal curve of urban sprawl, three archetypes of urban sprawl were noted, specifically the Early Urban Growth, the Constant Urban Growth and the Recent Urban Growth. Moreover, these urban sprawl trends match the western cities, eastern cities and central cities in China, respectively, in terms of their urban spatial configurations, socioeconomic characteristics and historical backgrounds. 3) In the spatial dimension, the spatial configuration of urban areas in Beijing belongs to the typical satellite town model, while the urban spatial configuration of Guangzhou and the surrounding towns represents a megalopolis. In summary, since 2000, China's urban areas have expanded at some rates, with an oscillation produced by international financial and policy changes, and other factors.

This paper aims to extract the series of urban extent in China and apply them to analyze the urban sprawl trends of China, instead of exploring the best method. Therefore, this paper did not perform the experiment at the cluster level. At the same time, we only assessed the accuracy of the result in 2000 using LULC data of 2000 in China, due to the lack of the reference urban map data. However, since it spans a long term from the reference year (2010) to the 2000 target year, the accuracy of the simulation result in the other target years should be higher than the one in 2000. Since this method processes NTL data at the regional level, the shape of the boundary for the image segmentation is arbitrary. In this study, using the administrative boundaries to divide the NTL units ensures the efficiency and flexibility of this method for practical situations. As a matter of fact, applying the boundary at the smaller scale can further improve the precision of the result. At the same time, this paper did not perform the experiment at the cluster level. This paper aims to extract the series of urban extent in China and apply them to analyze the urban sprawl trends of China, instead of exploring the best method.

In order to apply this method in other regions, two factors have to be confirmed: 1) the regional auxiliary data. This study use LULC data from the Resources and Environment Data Center of the Chinese Academy of Science in China as the auxiliary data to obtain the regional optimal thresholds using Greedy Search Method. In other regions, the regional LULC data from local official departments may be inaccessible. However, many researchers have been conducting studies on the global land-use and land-cover mapping with high resolution [68–70]. The global LULC products can be applied to this method as long as it is verified with local statistics data in other study areas; 2) the status of regional urbanization. This study select PIFs based on the typically irreversible nature of urbanization in recent years in China. According to the UN’s World Urbanization Prospects, the process of global urbanization has experienced rapid growth [71]. By 2014, approximately 54% of residents had lived in urban areas, and this proportion is expected to increase to 66% in 2050. And China, as the world’s largest developing country, is an important growth pole for the future of global urbanization. In only a small part of the world, urbanization is in a state of reverse growth. Most of them are located in the low-fertility countries of Europe and Asia [71]. Therefore, the method we proposed cannot be applied to these regions. And this is the issue we are currently studying—how to further improve the universality of the proposed method.

At present, the application of NPP/VIIRS has become a new hotspot in the field of NTL research because of the higher spatial resolution, higher temporal resolution, and continuous updating of NPP/VIIRS. However, the proposed method is not currently compatible with NPP/VIIRS. Many problems still remain unsolved, e.g., data format, inconsistency, and saturation. It is worth studying how to integrate NPP/VIIRS with DMSP/OLS and apply them to the proposed method in the future.

The improved method presented in this study makes rapid urban land monitoring over long time periods based on time-series NTL data possible by solving the problems associated with the temporal and spatial inconsistency of the NTL data. The results of this study show that the trends in urban sprawl agree well with the spatial configuration, socioeconomic characteristics and historical background of cities in China. Therefore, this study provides a high-precision urban extent dataset for studies of long-term global urban monitoring and land urbanization and shows that NTL data still have great potential for application in extracting macro-scale scenes for use in urban studies. The proposed urban extent dataset is available for download and can provide reference data and support for future studies of urban areas and urban planning.

## Supporting information

S1 File The time series of urban extent in China underlying the findings described in manuscript. (ZIP)

S2 File The raw files of all figures contained in manuscript. (ZIP)
