# Supplementary figures and images for: A time series of urban extent in China using DSMP/OLS nighttime light data
Source: PLoS One. 2018 May 24;13(5):e0198189. doi: 10.1371/journal.pone.0198189 (PMC5993125; doi:10.1371/journal.pone.0198189)

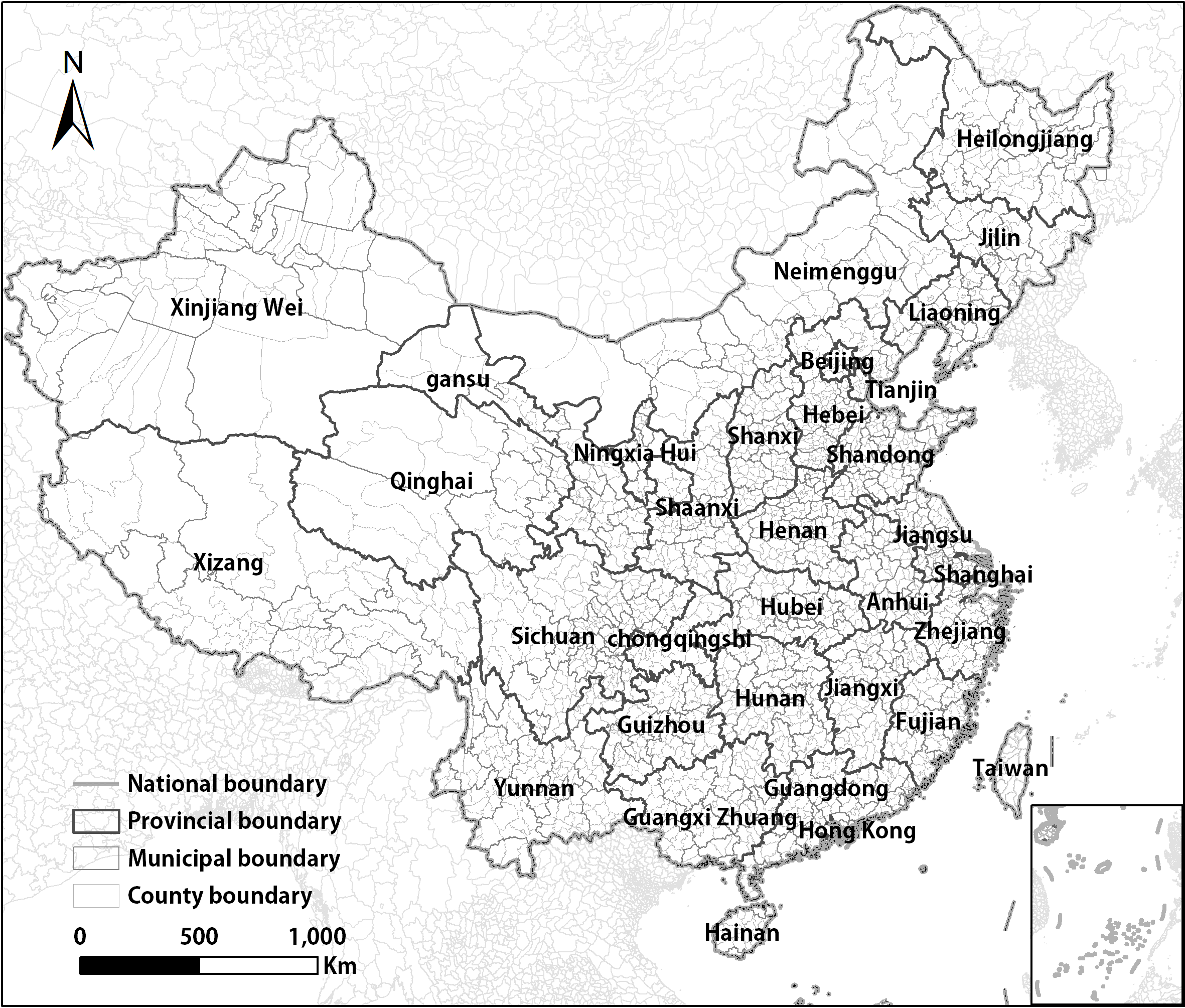

Supplement: S2 File — (ZIP) [file pone.0198189.s002.zip › Fig1.tif]

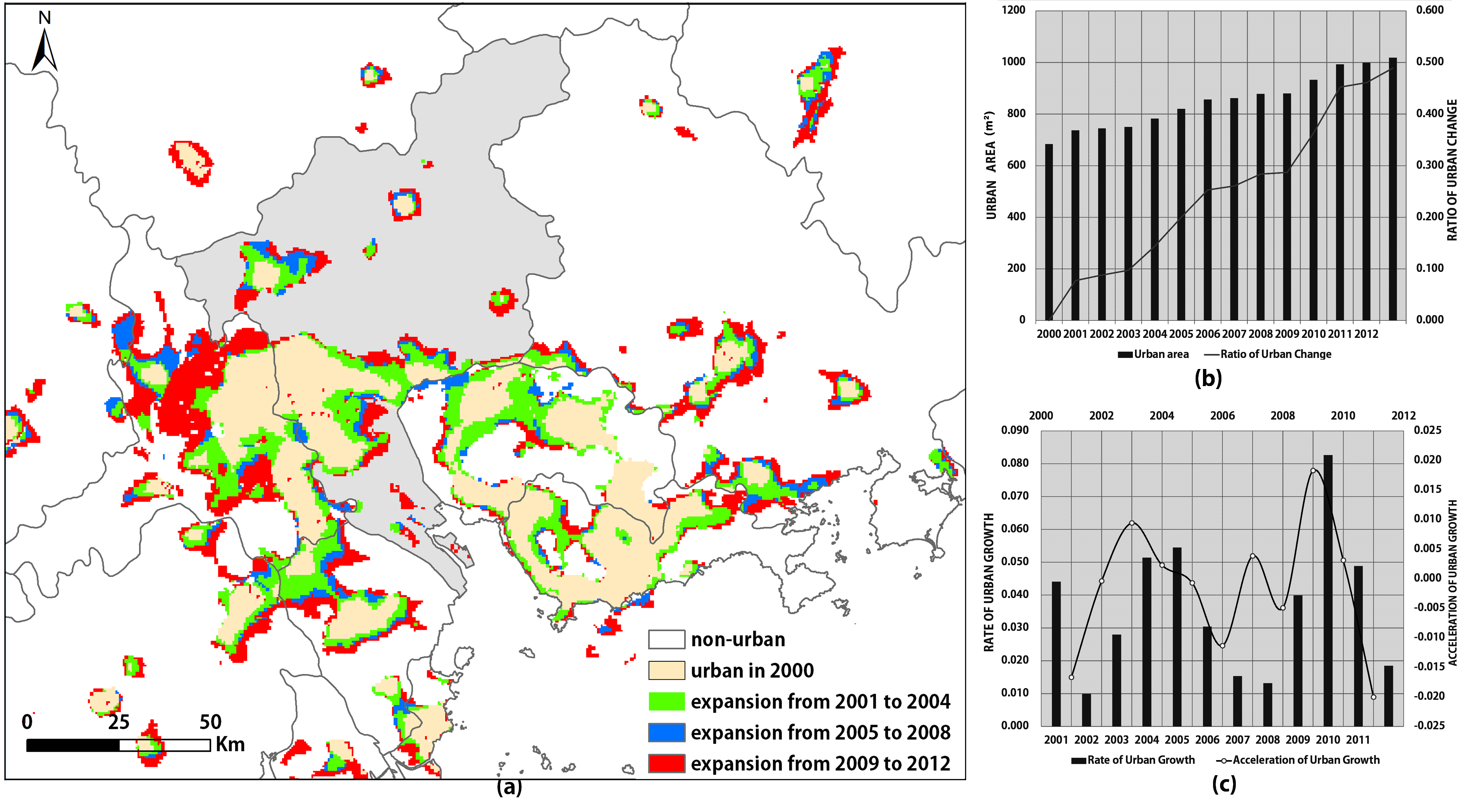

Supplement: S2 File — (ZIP) [file pone.0198189.s002.zip › Fig10.tif]

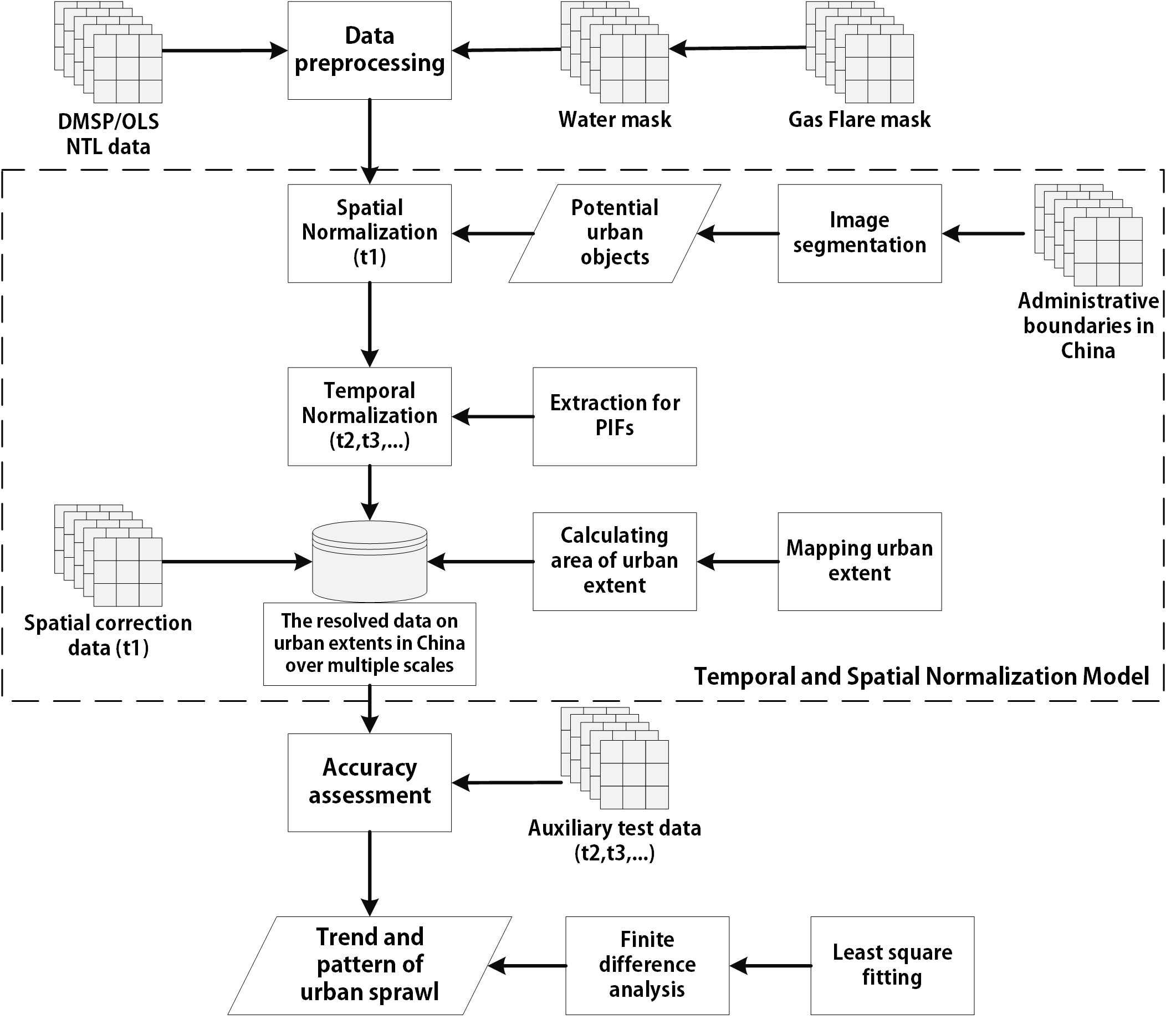

Supplement: S2 File — (ZIP) [file pone.0198189.s002.zip › Fig2.tif]

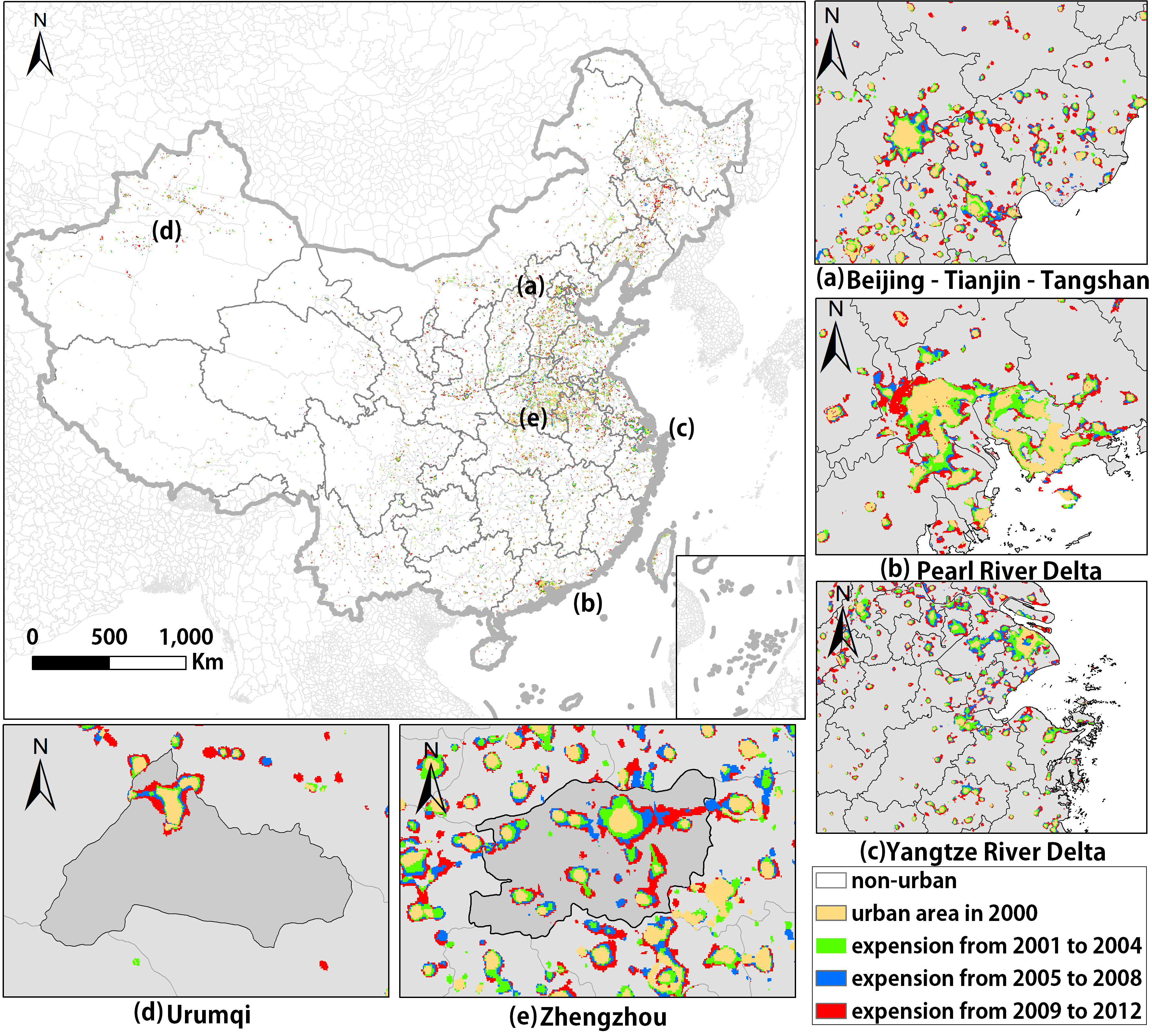

Supplement: S2 File — (ZIP) [file pone.0198189.s002.zip › Fig3.tif]

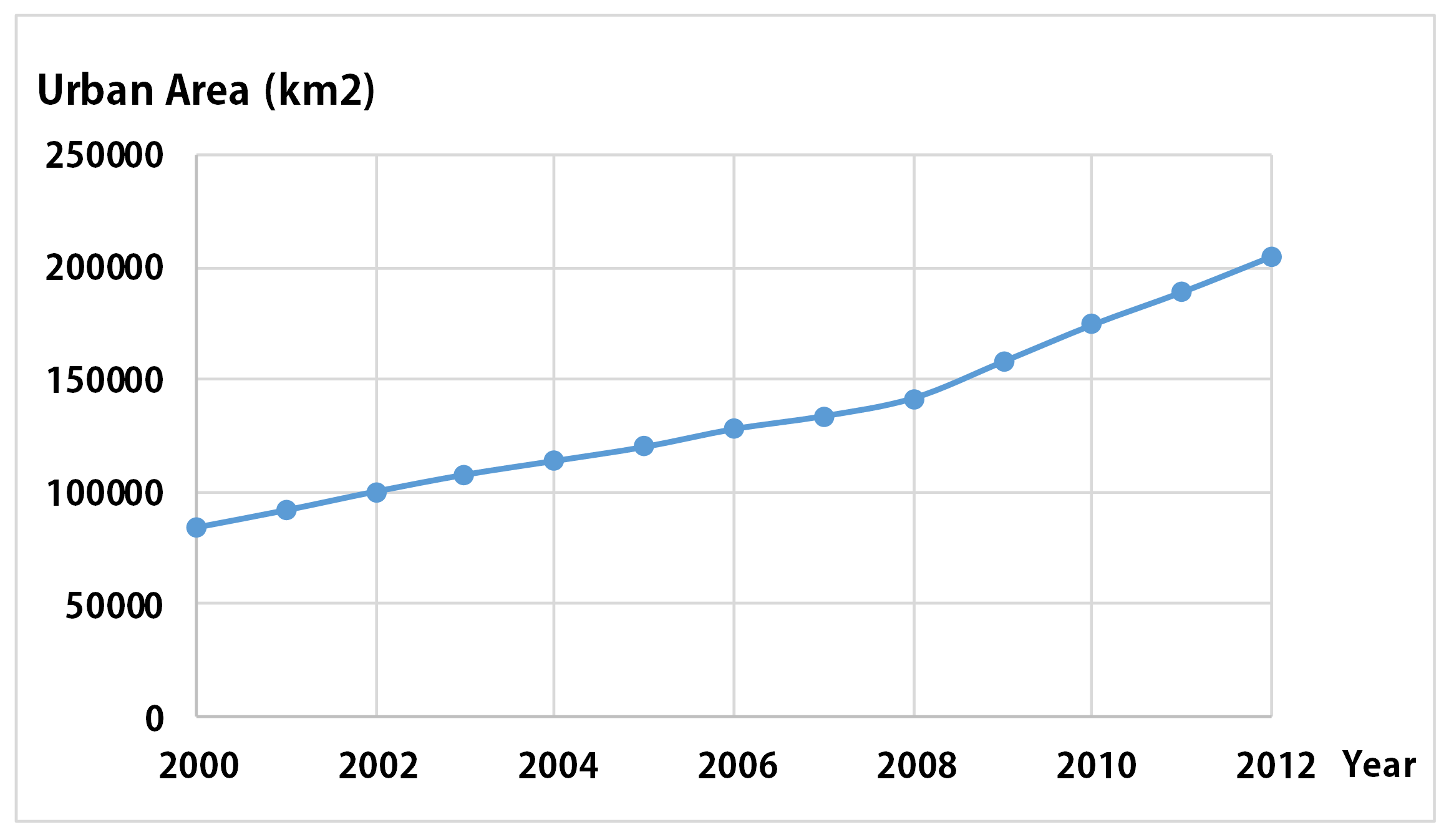

Supplement: S2 File — (ZIP) [file pone.0198189.s002.zip › Fig4.tif]

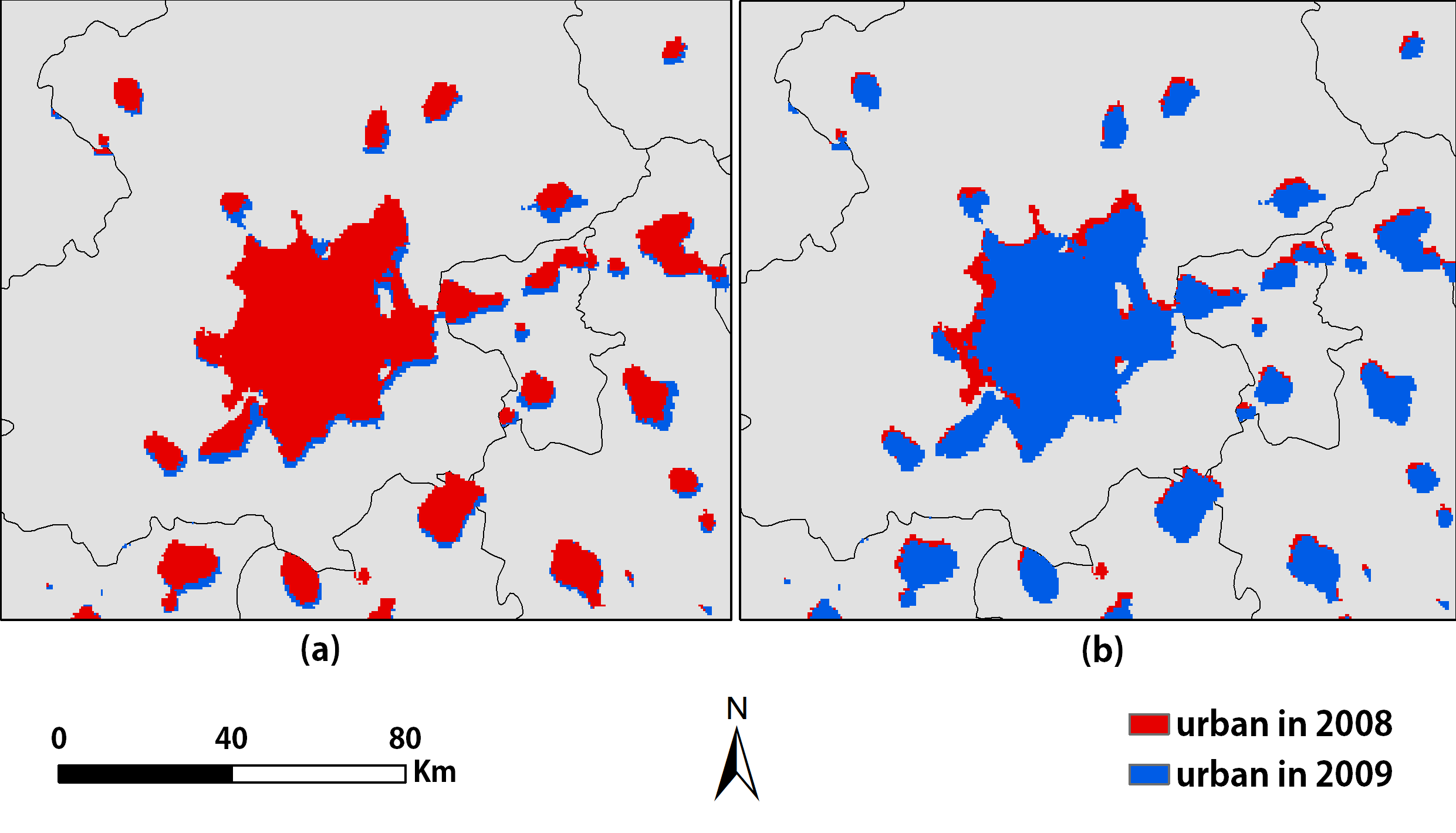

Supplement: S2 File — (ZIP) [file pone.0198189.s002.zip › Fig5.tif]

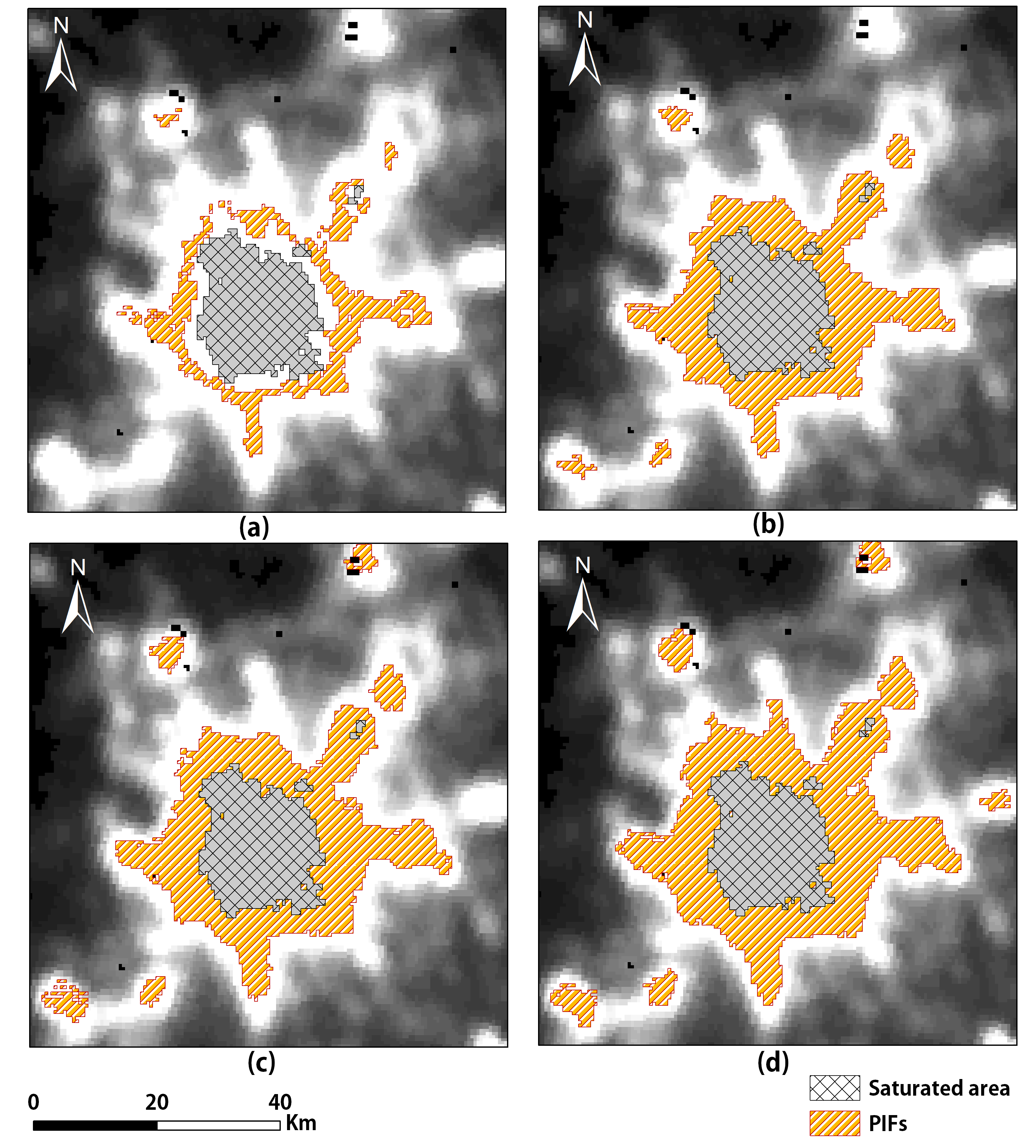

Supplement: S2 File — (ZIP) [file pone.0198189.s002.zip › Fig6.tif]

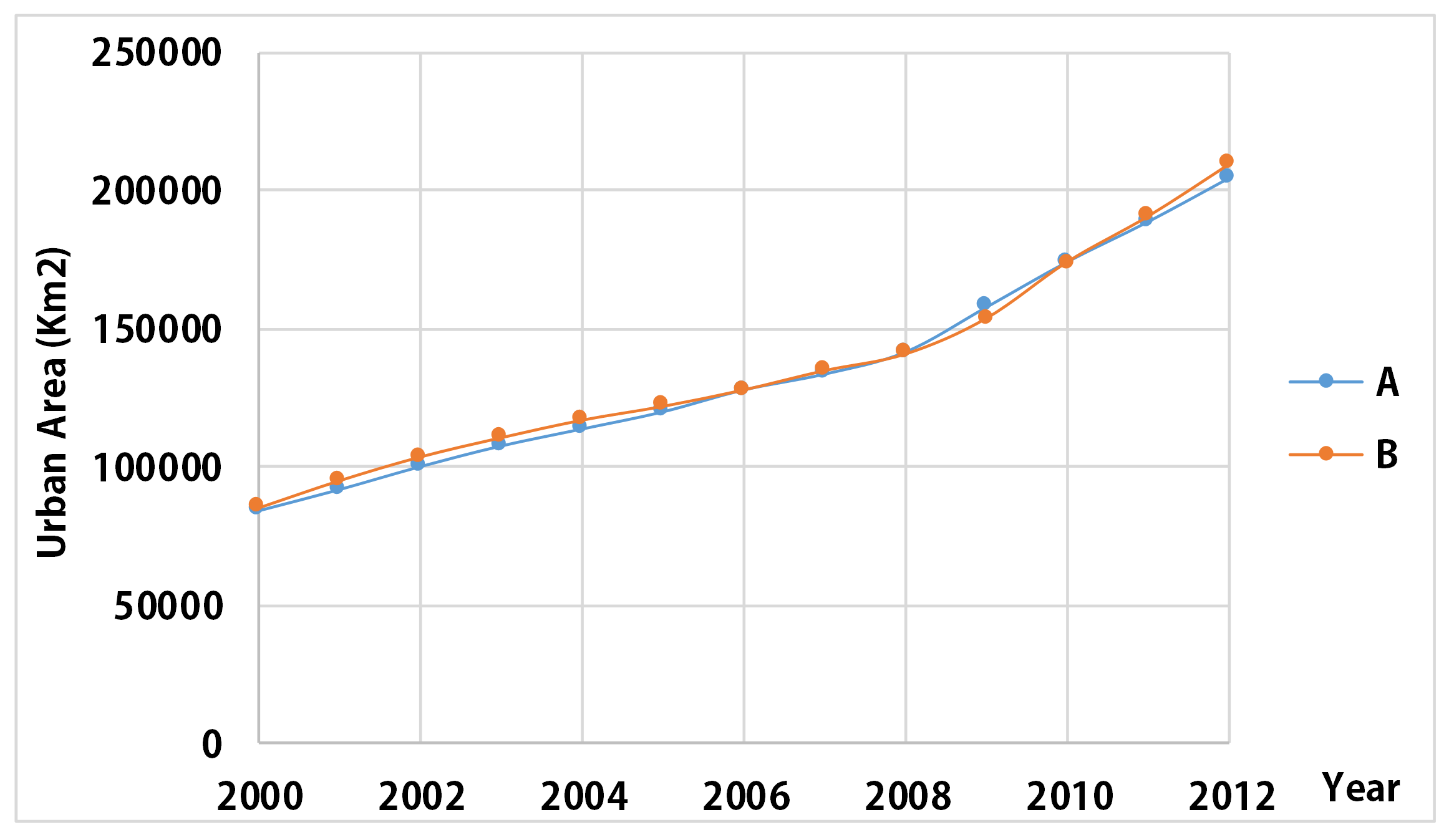

Supplement: S2 File — (ZIP) [file pone.0198189.s002.zip › Fig7.tif]

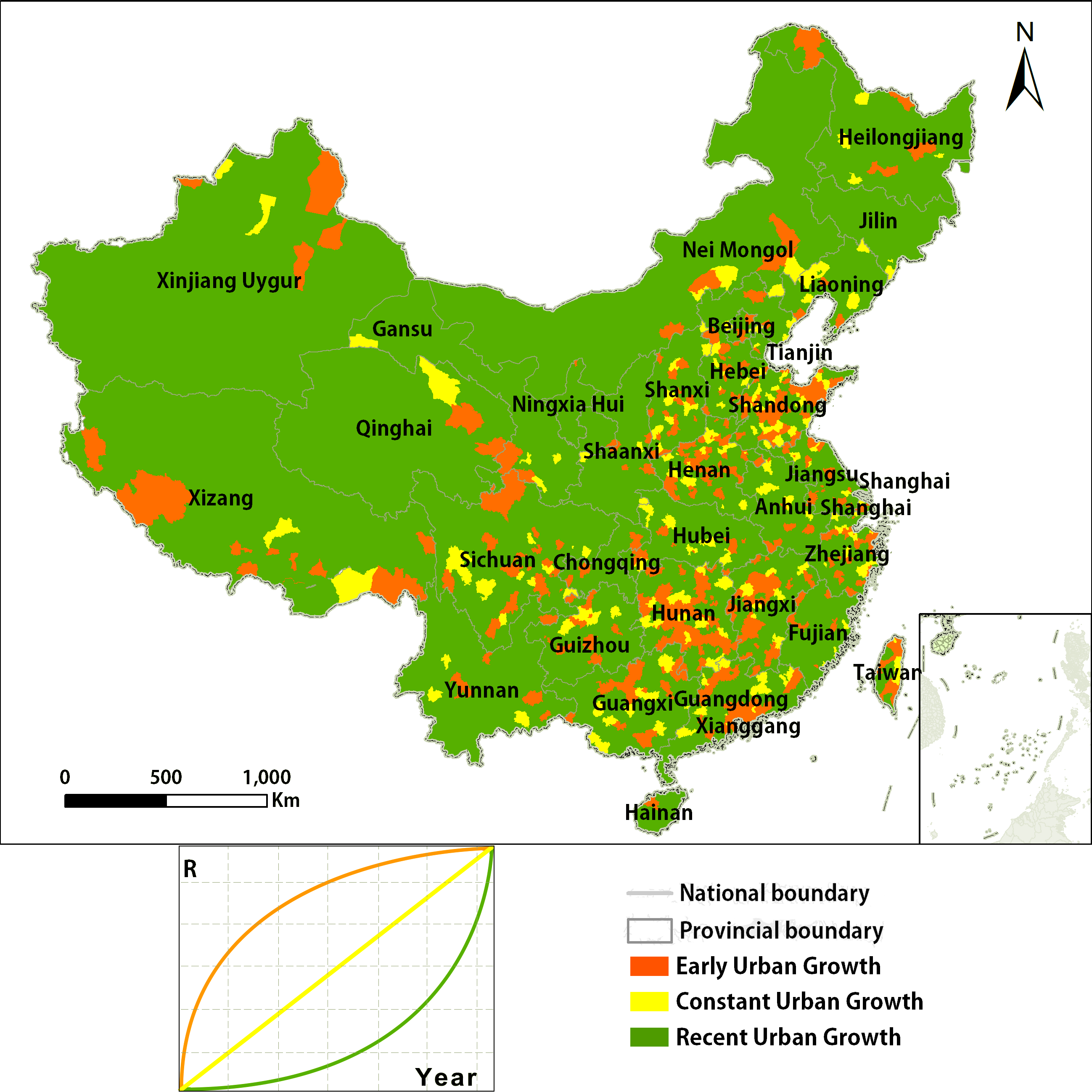

Supplement: S2 File — (ZIP) [file pone.0198189.s002.zip › Fig8.tif]

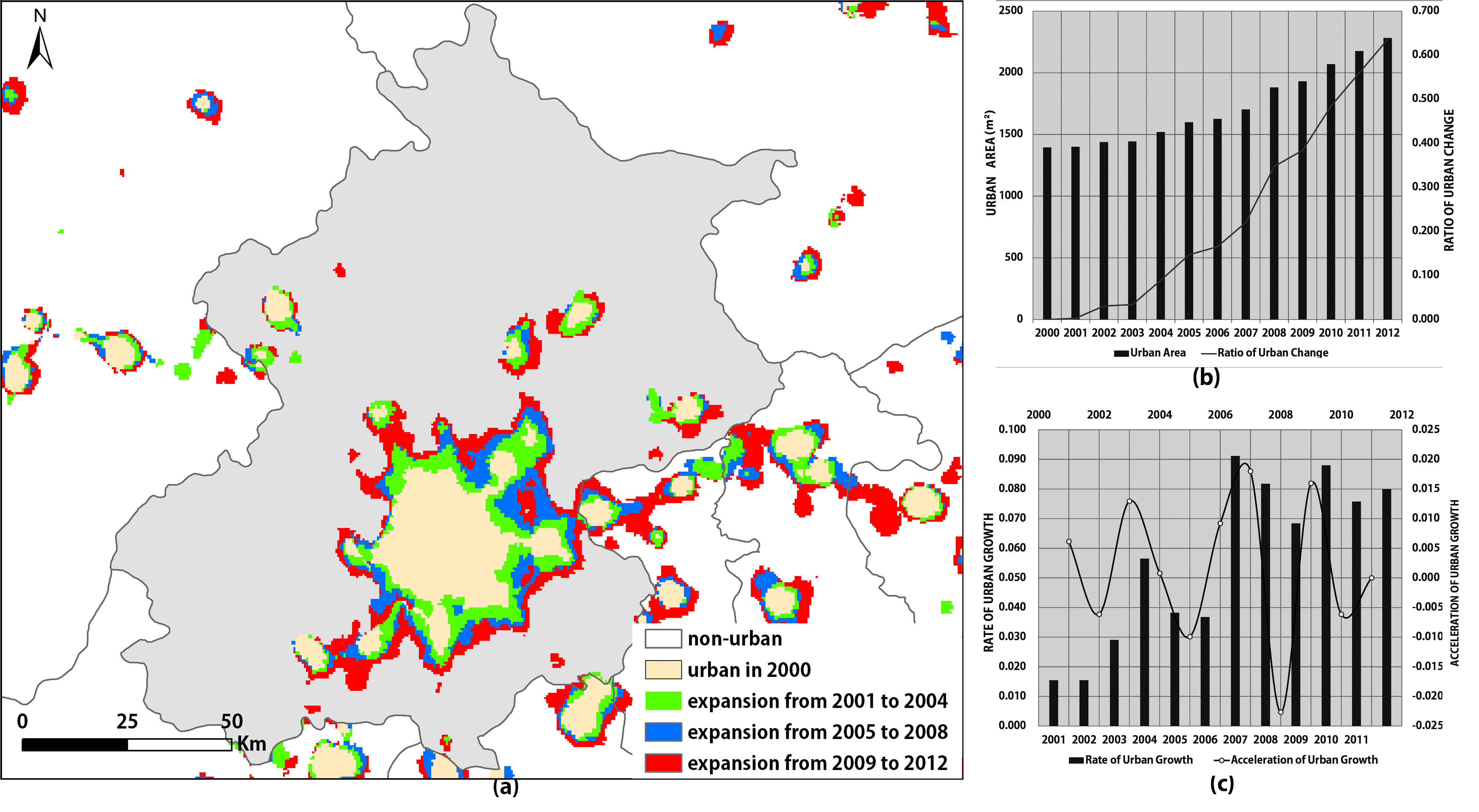

Supplement: S2 File — (ZIP) [file pone.0198189.s002.zip › Fig9.tif]
